# Anti-Inflammatory Therapeutic Mechanisms of Isothiocyanates: Insights from Sulforaphane

**DOI:** 10.3390/biomedicines12061169

**Published:** 2024-05-24

**Authors:** Solomon Habtemariam

**Affiliations:** Pharmacognosy Research & Herbal Analysis Services UK, University of Greenwich, Central Avenue, Chatham-Maritime, Kent ME4 4TB, UK; s.habtemariam@herbalanalysis.co.uk

**Keywords:** sulforaphane, Nrf2, antioxidant, anti-inflammatory, nuclear factor κB, signalling paradox

## Abstract

Isothiocyanates (ITCs) belong to a group of natural products that possess a highly reactive electrophilic −N=C=S functional group. They are stored in plants as precursor molecules, glucosinolates, which are processed by the tyrosinase enzyme upon plant tissue damage to release ITCs, along with other products. Isolated from broccoli, sulforaphane is by far the most studied antioxidant ITC, acting primarily through the induction of a transcription factor, the nuclear factor erythroid 2–related factor 2 (Nrf2), which upregulates downstream antioxidant genes/proteins. Paradoxically, sulforaphane, as a pro-oxidant compound, can also increase the levels of reactive oxygen species, a mechanism which is attributed to its anticancer effect. Beyond highlighting the common pro-oxidant and antioxidant effects of sulforaphane, the present paper was designed to assess the diverse anti-inflammatory mechanisms reported to date using a variety of in vitro and in vivo experimental models. Sulforaphane downregulates the expression of pro-inflammatory cytokines, chemokines, adhesion molecules, cycloxyhenase-2, and inducible nitric oxide synthase. The signalling pathways of nuclear factor κB, activator protein 1, sirtuins 1, silent information regulator sirtuin 1 and 3, and microRNAs are among those affected by sulforaphane. These anti-inflammatory actions are sometimes due to direct action via interaction with the sulfhydryl structural moiety of cysteine residues in enzymes/proteins. The following are among the topics discussed in this paper: paradoxical signalling pathways such as the immunosuppressant or immunostimulant mechanisms; crosstalk between the oxidative and inflammatory pathways; and effects dependent on health and disease states.

## 1. Overview of Chemistry and Biological Relevance

**Figure 1:**
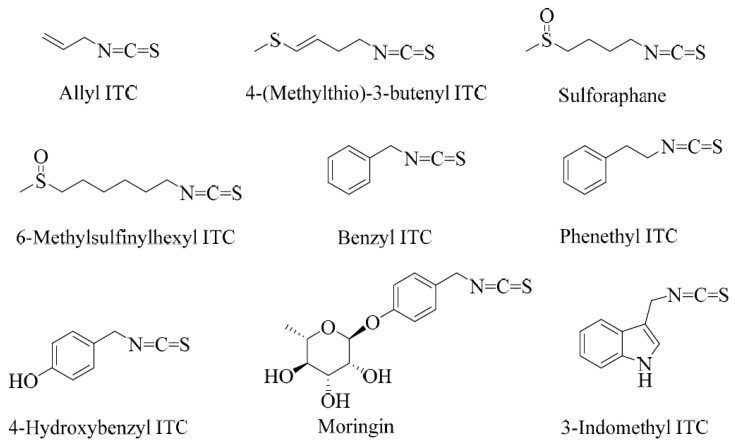
Examples of some common isothiocyanates from plant sources.

Sulforaphane is a small-molecular-weight sulphur-containing compound that belongs to a structural group of natural products called isothiocyanates (ITCs). Other examples of ITCs include benzyl, phenethyl, and allyl ITCs (Figure 1). The distinguishing feature of this class of compounds is the highly reactive electrophilic −N=C=S structural moiety, which undergoes several reactions in biological systems. The structural diversity of isothiocyanates in nature is represented by the side-chain R group (R-N=C=S), which can be made of aralkyls such as benzyl, 2-phenyl and 4-hydroxybenzyl, indoles such as indol-3-methyl or 4-hydroxyindol-3-ylmethy, or several aliphatic chain derivatives. Plants that produce ITCs store them in special cellular and subcellular sites in the form of precursor compounds called glucosinolates.

**Figure 2:**
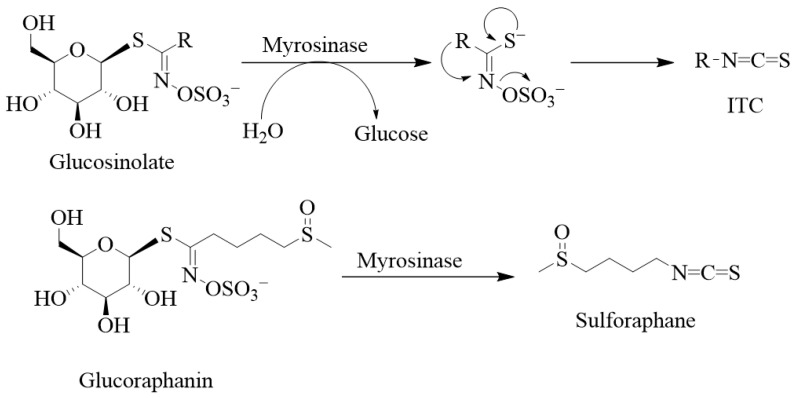
Degradation of glucosinolates and some examples of isothiocyanates. Glucosinolates are composed of the thiohydroximate-*O*-sulphonate group linked to glucose. Removing the glucose unit from glucosinolates by myrosinase leads to the unstable aglucon, meaning that thiohydroximate-*O*-sulphate is released. Hence, myrosinase catalyses the hydrolysis of these *S*-glucosides to give D-glucose and an aglycone fragment, and the aglycon further rearranges itself to give sulphate and products, mostly isothiocyanate. In the case of sulforaphane, the glucosinolate precursor is glucoraphanin.

Structurally, the glucosinolates are thiohydroximates that contain an *S*-linked β-glucopyranosyl and *O*-linked sulphate residues (Figure 2). As it has been said above for ITCs, structural diversity is based on the nature of the R group of either the alkyl, aralkyl, or indolyl side chains that derive from amino acids (e.g., phenylalanine, tryptophan, and methionine). Agerbirk and Olsen [1] reported in the year 2012 the existence of around 132 glucosinolates isolated from plants, and this number has been further increased over the past decade. Glucosinolates and their ITC products are generally considered defensive or protective chemicals against herbivores, insects, and pathogens [2,3]. Upon damage of the plant tissues, say by herbivores, glucosinolates encounter the enzyme myrosinase (β-D-thioglucosidase; EC 3.2.1.147), leading to their breakdown into various products such as nitriles, thiocyanates, and isothiocyanates, with the latter being the most stable. Other products known to derive from hydrolysis by the enzyme include epithionitriles, hydroxynitriles, oxazolidine-2-thiones, and indoles. Thus, the key to ITC production is that the relevant enzyme (myrosinase) and substrates (glucosinolates) are stored in plants in separate cellular compartments [4], and the hydrolysis reaction is only initiated upon cellular damage. The enzyme cleaves the thio-linked glucose from glucosinolates to form an unstable intermediate, thiohydroximate-*O*-sulphonate. The final product depends on a variety of factors, including the immediate pH, temperature, specifier proteins, and ferrous ions [5,6,7]. For example, a neutral or alkaline pH can spontaneously lead to ITC formation. As shown by Williams et al. [8] on myrosinase activity in *Lepidium sativum* and *Nasturtium officinale* seeds, a pH of less than 4 along with ferrous ions (Fe^2+^) and the epithiospecifier protein favour nitrile formation. Further variations in the degradation products also depend on the nature of the side chain.

Glucosinolate- or ITC-producing plants mostly belong to plants of the order Brassicales, which include families such as the Brassicaceae, the Capparaceae, and the Caricaceae. The Brassicaceae family, which is also called the family of cruciferous vegetables, has around 346 accepted genera and over 3000 species, including cultivated crops such as broccoli, cabbages, Brussel sprouts, cauliflowers, mustard seeds, etc. Sulforaphane is among the best investigated ITCs, and, biogenically, it is derived from the glucosinolate glucoraphanin (Figure 2). Broccoli is the best-known source of sulforaphane and the C3-C6 aliphatic chain, as the R group is commonly found in *Brassica* species. Allyl ITC is the main component behind the pungency of wasabi (Japanese horseradish; *Wasabia japonica*) and black mustard (*Brassica nigra*) seeds and is formed from its corresponding glucosinolate, sinigrin [9,10,11]. In contrast, white mustard (*Sinapis alba* L.) seeds have *p*-hydroxybenzyl ITC as a main component for their flavour and aroma. This is generated via a myrosinase action on the corresponding glucosinolate, sinalbin [12]. 4-(Methylthio)-3-butenyl ITC is a component of Japanese white radish (*Raphanus sativus*), while 6-methylsufinylhexyl ITC has been isolated in a good amount from wasabi [13]. Benzyl ITC has been isolated from papaya (*Carica papaya* Linn.) peel, pulp, and seeds [14] and phenethyl ITC from watercress (*Nasturtium officinale*) [15]. The sugar derivatives of hydroxybenzyl ITC are represented by moringin, isolated from *Moringa oleifera* and *M. stenopetala* [16].

The bioactivity and electrophilic nature of ITCs are attributes of the R−N=C=S structural skeleton, which undergoes several reactions in biological systems. The electron-deficient central carbon atom in such structure is susceptible to nucleophilic attacks by the electron-rich centre. For example, thiol groups such as glutathione can be added to ITC molecules. The implication of this reaction is huge and includes the generation of reactive oxygen species (ROS) by depleting glutathione, through a direct covalent reaction with the glutathione *S*-transferase (GST) of other thiol-containing biological molecules such as proteins and enzymes. Hence, sulforaphane is among the best-known compounds generating the oxidative stress-based induction of apoptosis in cancer cells. In fact, by far the most studied biological activity of isothiocyanates is in the cancer field, where sulforaphane from broccoli, among others, has been shown to prevent carcinogenesis [17,18,19] and induce apoptosis in cancer cells [20,21,22]. For example, sulforaphane suppresses the incidence of tumours in mice exposed to UV light [23]. Sulforaphane also suppresses cancer cell migration and invasion [24], prostate carcinogenesis and pulmonary metastasis [25], and carcinogenicity induced by cadmium [26]. The immunomodulatory effect of sulforaphane in cancer is also well established and includes boosting the activity of immune cells such as natural killer cells against cancer [25]. It augments natural killer cell- and antibody-dependent cellular cytotoxicity by enhancing the production of cytokines IL-2 and IFN-γ [27]. In B16F-10 melanoma-induced metastasis-bearing C57BL/6 mice, sulforaphane has been shown to enhance natural killer cell activity while also enhancing antibody-dependent cellular cytotoxicity in metastatic tumour-bearing animals [28]. It is also worth noting that electrophiles and oxidants are detoxified in the body by phase II metabolic enzymes such as GST and NAD(P)H:quinone oxidoreductase 1 (NQO1). Interestingly, the antioxidant and antimutagenic effects of ITCs, including sulforaphane, are associated with the induction of GST and NQO1 [29,30]. Not surprisingly, isothiocyanates have numerous other biological activities, such as antifungal and bactericidal [31,32,33,34] as well as antiparasitic [35] activities. In this paper, the anti-inflammatory mechanisms of sulforaphane are scrutinised by assessing all the publications sourced from the literature (Web of Science, PubMed, and ScienceDirect) until February 2024. 

## 2. Anti-Inflammatory Effects In Vivo

Readers should note that the present paper was designed to present a mechanistic overview of sulforaphane as an anti-inflammatory agent. This was achieved (see below) by assessing the in vitro studies in which sulforaphane had been shown to target inflammatory cells such as leukocytes, astrocytes, and endothelial, epithelial, and other cell types (Table 1, Table 2, Table 3, Table 4 and Table 5). With this in mind, it is worth highlighting that the anti-inflammatory effect of sulforaphane has further been confirmed through in vivo studies. These include experimental models in rats such as acetaminophen hepatitis [36] and hepatic ischaemia/reperfusion injury, where the expression of inflammatory mediators (cyclooxygenase-1 (COX-2), tumour necrosis factor-α (TNF-α), interleukin (IL)-6, and monocyte chemoattractant protein-1 (MCP-1)) and reactive oxygen species (ROS) production have been demonstrated to be inhibited. At the same time, the expression of the oxidative stress regulator transcription factor, the nuclear factor erythroid 2-related factor 2 (Nrf2), and its downstream gene/protein products (*NQO1*, haeme oxygenase-1 (*HO-1*), glutathione (*GSH*), catalase (*CAT*), and superoxide dismutase (*SOD*)) have been shown to be upregulated. Interestingly, the Nrf-2 inhibitor ML385 has been shown to reverse the observed anti-inflammatory and antioxidant effect [37]. Other rat models of inflammation in the liver where sulforaphane has shown positive results include non-alcoholic fatty liver disease [38] and sodium valproate-induced acute liver injury [39]. In a rat heart inflammation model, sulforaphane ameliorated doxorubicin-induced chronic heart failure [40] and reduced fibrosis and the scores of post-myocardial infarction associated with an increased HO-1 level [41], as well as the positive anti-inflammatory score in acrolein-induced cardiomyopathy [42], the cardiac ischaemia/reperfusion model [43], and cuprizone-induced cardiotoxicity [44]. Ischaemia/reperfusion injuries in rats have been further used to demonstrate the anti-inflammatory activity of sulforaphane in various organs, including the lungs [45] and retina [46,47]. Inflammation associated with diabetes in rats has been extensively used in sulforaphane studies and includes efficacy in diabetic neuropathy [48], a diabetic model of renal inflammation [49], experimental diabetic peripheral neuropathy in rats [50], and streptozotocin (STZ)-induced diabetic rats [51]. Further research on renal inflammation in rats has included folic acid-induced acute renal injury [52] and cisplatin-induced nephropathy where the expression of pro-inflammatory cytokine (TNF-α) and nuclear factor κB (NF-κB) were suppressed [53]. Other inflammation models in rats for sulforaphane research were cancer-induced bone pain [54], carrageenan-induced oedema [55], the traumatic haemorrhagic shock model [56], chromium-induced lung injury [57], arsenic-induced nephrotoxicity [58], ioversol-induced nephropathy [59], the neuroinflammation and spatial learning model [60], age-related renal injury in rats [61], anti-nociceptive and anti-inflammatory effects on a sciatic endometriosis rat model [62], and chronic renal allograft dysfunction [63].

Most research papers on the anti-inflammatory effect of sulforaphane in vivo are based on studies using mice experimental models. These include the classic anti-inflammatory in vivo model using carrageenan-induced oedema neuropathic pain [163], acrylamide-induced neuropathy [164], collagen-induced arthritis [79,165], osteoarthritis [166], adjuvant-induced chronic pain [167,168], chronic constriction injury-induced neuropathic pain [169], and acute gout [98] models. Other experimental models of inflammation employing mice are the demonstration of the anti-inflammatory potential of sulforaphane in lung diseases using cigarette smoke-induced alveolar damage [66], cigarette smoke-exposed asthmatic mice [170], bleomycin-induced pulmonary fibrosis [171], ovalbumin (OVA)-sensitised and cigarette smoke-induced airway inflammation [172], chlorine-induced airway hyper-responsiveness [173], haemorrhagic shock-induced lung injury [174], OVA-induced chronic allergic airways [175], lipopolysaccharide (LPS)-induced acute lung injury [176], respiratory syncytial virus (RSV)-induced bronchopulmonary inflammation [177], and the pulmonary arterial hypertension model [178].

Mice models of gut inflammation have been effectively used to show the anti-inflammatory effect of sulforaphane, such as those using dextran sodium sulphate (DSS)-induced gut inflammation [73,179], DSS-induced ulcerative colitis [118,180,181], high-fat high-cholesterol diet-induced gut inflammation [182], 5-fluorouracil-induced intestinal injury [183], necrotizing enterocolitis [184], gut inflammation associated with bladder cancer [185], the genetic model of intestinal polyps [186], and the genetic model of gastrointestinal dysfunction [187]. Anti-inflammatory effects in the liver of mice have been shown for sulforaphane using experimental models including the following: carbon tetrachloride-induced acute liver injury [188]; high-fat diet-induced non-alcoholic fatty hepatic steatosis and liver disease [189,190,191]; high-fat diet-induced [192,193]; LPS-induced acute liver injury [128,194,195,196]; hepatic ischaemia/reperfusion injury [197]; sickle cell disease model [198]; and cadmium-induced hepatotoxicity [199]. Inflammation associated with diabetes in mice has been effectively ameliorated by sulforaphane, as shown in high-fat diet- or STZ-induced diabetes and cardiomyopathy [200,201], *db*/*db* diabetic mice cardiomyopathy [202], diabetic cardiomyopathy in both type 1 and type 2 diabetes [203,204], high-fat diet-induced diabetes [205,206], *ob/ob* diabetic mice [90], obesity- and type 2 diabetes-associated pain [207], STZ-induced diabetes [208,209], STZ-induced diabetic nephropathy [210], type 1 diabetic OVE26 mice [211], and diabetes-induced vascular inflammation and pathogenesis [212]. Using a high-fat diet model, the anti-inflammatory effects of sulforaphane in mice have been further shown in [213,214] as well as in American diet-induced inflammation [215], TNF-α-induced vascular inflammation [121,122], and high-fat diet-induced obesity [196] models.

Infection models in mice have been used to show the anti-inflammatory effect of sulforaphane as demonstrated using helicobacter pylori infection [216], SARS-CoV-2 infection [217], LP-BM5 leukaemia retrovirus infection [218], and microcystin-LR (MC-LR)-induced inflammation [219]. These effects have been further validated using experimental autoimmune encephalomyelitis [220], autoimmune encephalomyelitis [74,221], LPS-induced acute inflammation [222], necrotizing enterocolitis [223], respiratory syncytial virus (RSV)-induced bronchopulmonary inflammation, epithelial injury [177], and LPS-induced endotoxemia [224] models.

Other anti-inflammatory effects of sulforaphane in mice have been based on ultraviolet B (UVB)-induced skin inflammation [225,226], the genetic model of muscular dystrophy [227,228], ischemia/reperfusion-induced muscular injury [229], ischaemia/reperfusion injury and cardiac allograft vasculopathy [127], vascular remodelling in hypoxic pulmonary hypertension [230], aged mice [231], angiotensin II-induced renal inflammation and injury [232], ischaemia/reperfusion injury [233], hypoxia-induced cardiomyopathy [234], the genetic model of kidney disease [235], retinitis Pigmentosa [236], atopic dermatitis [237], UV radiation-induced inflammation [238], acute exhaustive exercise-induced organ damage and inflammation [239], radiation-induced skin damage [240], oxazolone-induced chronic itch model [241], and acute pancreatitis in mouse [158] experimental models.

In the CNS domain, experimental models in mice for the anti-inflammatory effect of sulforaphane have included spinal cord injury [242,243], depression-like behaviour [244], LPS-induced depression-like behaviours [245,246,247], LPS-induced spatial learning and memory dysfunction [248], the transgenic model of Alzheimer’s disease [249], the genetic model of autism [250], contusion spinal cord injury [251], MG132-mediated spatial memory loss [252], LPS-induced depressive disorder [253], and the platelet aggregation and thrombus-associated cerebral microcirculation [254]. Some experimental models employing rabbits have also been used to demonstrate the anti-inflammatory effect of sulforaphane [255,256,257,258]. 

## 3. Anti-inflammatory Studies In Vitro

The effects of sulforaphane as an anti-inflammatory agent in vitro have been shown in cellular models using leucocytes (Table 1), astrocytes and glial (Table 2), endothelial (Table 3), epithelial (Table 4), and many other (Table 5) cell types. These effects are attributed to the suppression of the expression or activity of the various inflammatory mediators described below.

### 3.1. Anti-inflammatory Effect of Sulforaphane through the Suppression of Pro-Inflammatory Cytokines and Chemokine Production

Pro-inflammatory cytokines such as IL-1β, TNF-α, IL-6, and IL-8 play a pivotal role in the pathogenesis of various chronic inflammatory diseases. Numerous therapeutic approaches using antibodies target these cytokines or their receptors. Good examples of these agents are marketed drugs for chronic inflammatory diseases, including the following: adalimumab, infliximab, and certolizumab against TNF-α; rilonacept, anakinra (receptor antagonist), and canakinumab against IL-1; and tocilizumab and siltuximab against IL-6. On the other hand, small-molecular-weight inhibitors target the signalling pathways of these cytokines’ production, such as those induced by LPS, pro-inflammatory cytokines, ROS, or other inducers. The expression of such cytokines by a variety of agents has been shown to be suppressed by sulforaphane in alveolar macrophages [70], peritoneal macrophages [72], adipose tissue macrophages [75], bone marrow-derived macrophages [73], THP-1 or peripheral blood mononuclear cell (PBMC)-derived macrophages [75,77,80,81,82,86,99,100], murine RAW264.7 cells [89,90,91,93,95,97], dendritic cells [74,76], and T-cells [101]. Similarly, sulforaphane has been shown to suppress the expression of pro-inflammatory cytokines in microglial BV2 cells [103,110,113,115], N9 murine microglial cells [105], senescent astrocytes [108], primary co-cultures of rat microglial and astroglial cells [111], and Müller cells of the retina [51]. Other inflammation models in vitro where cytokine production has been suppressed by sulforaphane include mast cells [102], endothelial cells such as human umbilical vein endothelial cells (HUVECs) [121,122], saphenous vein endothelial cell [127], and transformed endothelial cells such as ECV304 [124]. Similarly, the upregulation of cytokines’ production in epithelial cells has been shown to be suppressed by sulforaphane, including in Caco-2 [135], human lung epithelial cells (BEAS-2B) [136,143,145], and primary mouse tracheal and human bronchial epithelial cells [139]. Several other cell types under inflammatory conditions have also responded to sulforaphane to downregulate the expression of pro-inflammatory cytokines [147,148,151,155]. On the other hand, the expression of anti-inflammatory cytokines such as IL-10 is promoted by sulforaphane, as shown in senescent astrocytes [108], the LPS-activated C6 astrocyte cell line [116], and human monocyte-derived dendritic cells [76].

Moreover, in macrophages, the pro-inflammatory M1 marker’s morphology and genes (associated with pro-inflammatory cytokine production) are suppressed by sulforaphane, while it promotes the M2 marker genes associated with the anti-inflammatory mechanism [73,77,85,99]. In addition to IL-8, other chemokines’ expression, especially in the lung inflammation model, has been shown to be suppressed by sulforaphane. For example, the induced expression of MCP-1 [131,143] and MCP-1 and chemokine (C-X-C motif) ligand 1 (*CXCL-1*) [136] in airway epithelial cells is inhibited. Also, the expression of MCP-1 in HUVECs has been shown to be reduced by sulforaphane [119,121,122]. Hence, while multiple mechanisms may be implicated (see the following sections), the major anti-inflammatory mechanism of sulforaphane is attributed to the inhibition of the expression of pro-inflammatory cytokines and chemokines.

### 3.2. Anti-Inflammatory Effect of Sulforaphane through the Inhibition of the Expression of Adhesion Molecules

The major impact of pro-inflammatory cytokines such as IL-1 and TNF-α lies in their ability not only to induce the expression of other inflammatory cytokines or mediators but also the expression of key cell-surface adhesion molecules, primarily on endothelial and leucocyte cell surfaces. Over the last four decades, numerous research studies have shown that the expression levels of intracellular adhesion molecule 1 (ICAM-1), vascular cell adhesion molecule 1 (VCAM-1), and E-selectin on endothelial cell surfaces provide a good indication of the anti-inflammatory potential of therapeutic agents. In this connection, the endothelial cell surface expression of adhesion molecules such as ICAM-1 [124], VCAM-1 [121,122,129], ICAM-1 and VCAM-1 [119,120], and E-selectin and VCAM-1 [128] has been shown to be inhibited by sulforaphane. This is also evident in other cell types, including retinal pigment epithelial cells, where the suppression of the expression of ICAM-1 [134] and, in vascular smooth muscle cells (VSMCs), ICAM and VCAM [149] or VCAM-1 [150] has been observed. Similarly, the induced expression of ICAM-1 in epithelial cells by a variety of inflammatory mediators has been shown to be ameliorated [120,121,122,134]. Consequently, monocyte adhesion to activated endothelial cells has also been shown to be inhibited by sulforaphane [120,121,122]. Hence, the numerous in vivo anti-inflammatory effects of sulforaphane described in Section 2 are an attribute of both the suppression of the level of expression of pro-inflammatory cytokines as well as their effect on inflammatory cascades resulting from the reduction in adhesion molecules’ expression.

### 3.3. Anti-inflammatory Effect of Sulforaphane through the Suppression of COX-2 Expression

Classical anti-inflammatory compounds such as aspirin, indomethacin, ibuprofen, and diclofenac as well as the newer generation of selective COX-2 inhibitors (e.g., celecoxib) target the enzymatic activity of COX-2, while others, including steroidal anti-inflammatory agents, suppress the induced expression of COX-2. In the latter case, the expression of COX-2 has been shown to be suppressed by sulforaphane in activated peritoneal macrophages [72], PBMC [78], THP1 or PBMC [85], RAW264.7 cells [89,93,94,96,97], BV2 microglial cells [110,115], and mammary epithelial cells [130,140]. Other cells in which the expression of COX-2 has been shown to be suppressed by sulforaphane under inflammatory conditions include vascular smooth muscle cell lines [150], neuroblastoma SH-SY5Y cells [161], and rheumatoid arthritis synovial fibroblasts [157]. Hence, some of the anti-inflammatory effect of sulforaphane can be attributed to a reduction in expression of the key enzyme COX-2, thereby inhibiting the production of pro-inflammatory prostaglandins. Unlike COX-1, which is constitutively expressed and involved in normal physiological functions such as gastrointestinal tract (GIT) protection, targeting the inflammation-mediated or -induced expression of COX-2 by therapeutic agents avoids the general side effect of non-selective COX inhibitors.

### 3.4. Anti-inflammatory Effect of Sulforaphane through the Inhibition of iNOS Expression

Like COX-2, inducible nitric oxide synthase (iNOS) is an enzyme isoform associated with the inflammation-induced expression of inflammatory mediators, leading to nitric oxide (NO) overproduction. Inhibitors of iNOS have, thus, been sought after to treat inflammatory diseases [259,260]. The pro-inflammatory mediator (e.g., LPS and cytokines)-induced expression of iNOS has been shown to be suppressed by sulforaphane, such as in peritoneal macrophages [71], bone marrow-derived macrophages [72], stimulated RAW264.7 cells [89,90,91,92,93,96,97], PMBC-derived macrophages [78,99], BV2 microglial cells [110], astrocytes [116], mammary epithelial cells [130], neuroblastoma SH-SY5Y cells [161], C2C12 myoblasts [147], and vascular smooth muscle cells (VSMCs) [150]. With Macrophages being known to be factories of the NO which contributes to both oxidative stress and inflammation, some of the observed effects of sulforaphane are likely to be attributed to the inhibition of inflammation-associated iNOS expression.

### 3.5. Anti-Inflammatory Effect of Sulforaphane through the Inhibition of Inflammation-Associated Oxidative Stress

It is also worth noting that sulforaphane is widely known for its antioxidant effect primarily via the activation of the transcription factor Nrf2 (Table 1, Table 2, Table 3, Table 4 and Table 5). Induced ROS production in various cellular models has been shown to be suppressed by sulforaphane, including in neutrophiles [64], murine bone marrow-derived macrophages [72], PBMC [78], RAW264.7 cells [88,93], human lung epithelial cells (BEAS-2B) [136], N9 murine microglial cells [105], primary astroglial rat cultures or mouse cerebral cortices [106], primary cultures of cortical astrocytes from newborn pig brains [107], BV2 microglial cells [110], HUVECs [117], human brain endothelial cell [128], primary cultures of cerebral microvascular endothelial cells [107], mammary epithelial cells [130], airway epithelial [132,144], retinal pigment epithelial (ARPE-19) cells [133], Caco-2 cells [135], human lung epithelial cells (BEAS-2B) [136], primary mouse and tracheal and human bronchial epithelial cells [139], and human airway epithelial (NCI-H292) cells [144]. Readers should bear in mind that these are selective examples of sulforaphane’s effect on the amelioration of ROS production, specifically associated with inflammation experimental models. As described in Section 4, this antioxidant activity associated with inflammation is coupled with the induction of antioxidant defences through the Nrf2 signalling pathway (Table 1, Table 2, Table 3, Table 4 and Table 5).

## 4. Mechanistic Overview of the Anti-Inflammatory Effect of Sulforaphane

Mirroring the in vivo investigations, mechanistic studies on sulforaphane have been largely performed in vitro using various inflammatory models. The Nrf2-dependent and Nrf2-independent pathways of the key mechanisms are discussed below.

### 4.1. Induction of Nrf2

**Figure 3:**
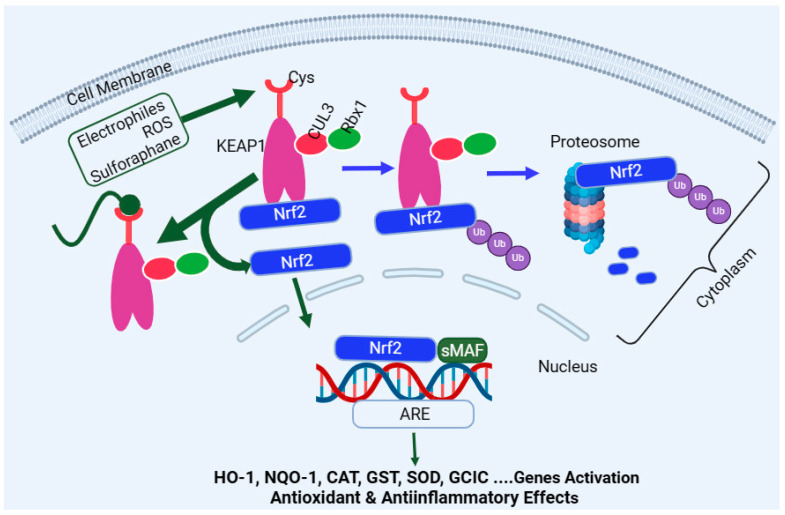
Overview of the Nrf2 activation pathway. Nrf2 is inactivated under normal physiological condition through binding to KEAP1, leading to its proteasomal degradation. Reactive oxygen species, oxidative stress, or electrophiles such as sulforaphane can inactivate KEAP1 via interaction with redox-sensitive cysteine (Cys) residues. This allows the newly formed Nrf2 to be free to translocate to the nucleus and associate with sMAF to bind with the ARE domain of the DNA, leading to activation of target genes such as HO-1, NQO-1, CAT, GST, SOD, and GCLC. Abbreviations: sMAF, small musculoaponeurotic fibrosarcoma; ARE, antioxidant response element; CUL3, cullin-3; GCLC, glutamate–cysteine ligase catalytic; NQO1, NADPH quinone oxidoreductase enzyme; SOD, superoxide dismutase; HO-1, haeme oxygenase-1; CAT, catalase; and GST, glutathione *S*-transferase. This figure has been generated using Biorender.

Encoded by the *Nfe2l2* gene, Nrf2 is a master regulator of multiple antioxidant enzymes such as HO-1, NQO1, CAT, SOD, glutathione peroxidase (GPx), GST, therodoxins, and glutamate–cysteine ligase (GCL). In the latter case, GCL is a heterodimer of the GCL catalytic subunit (GCLC) and the GCL modifier subunit (GCLM). The target genes regulated by the Nrf2 pathway are far more than antioxidant enzymes and include detoxification enzymes, DNA repair enzymes, and molecular chaperone proteins. The activation or upregulation of the Nrf2 level leads to cytoprotection under stress conditions through various mechanisms, including the removal of ROS and oxidative stress, as well as anti-inflammatory and anti-apoptosis mechanisms. On this basis, Nrf2 is considered to be an evolutionarily conserved defence mechanism for a wide range of living organism to defend themselves against oxidative damage and xenobiotics [261]. Under normal physiological conditions, the Nrf2 in the cytoplasm binds to its negative regulator, the Kelch-like epichlorohydrin-associated protein (KEAP1), to form complexes. KEAP1 is the recognition site for redox-dependent CULLIN 3 (Cul3)-based (or CUL3–ring-box 1 (Rbx1)-containing) E3 ubiquitin ligase, which degrades Nrf2 (Figure 3)—i.e., KEAP1 acts as an adaptor protein for ubiquitin ligase, which is responsible for the ubiquitylation and subsequent degradation of Nrf2 through the proteasome (26s) system [262,263]. This system is collectively called the CULLIN-RING ubiquitin ligases complex, where CUL3 serves as a scaffold protein which forms a complex of E3 ligase with Rbx1 to recruit a cognate E2 enzyme. As one would expect, high levels of Nrf2 or Nrf2-target genes are observed in *KEAP1* knockout mice and give these animals resistance to xenobiotics’ toxicity [264]. On the other hand, Nrf2-deficient mice have been shown to be susceptible to cigarette smoke-induced emphysema [265], hyperoxic lung injury [266], and pulmonary fibrosis [267]. One common activation pathway of Nrf2 is oxidative stress, ROS, or electrophiles, which directly interreacts with KEAP1 by oxidising or alkylating its cysteine residues, which are required for Nrf2 binding (Figure 3). This inactivates KEAP1 and removes the negative regulator of Nrf2 (removal of ubiquitination), leading to Nrf2 stabilisation and translocation into the nucleus to regulate target genes’ expression [268,269]. Upon entering the nucleus, Nrf2 heterodimerises with the small musculoaponeurotic fibrosarcoma (sMaf) protein family, which allows its binding to the antioxidant response element (ARE) sequence in the promoter regions of various target genes [270]. In addition to KEAP1, various protein kinases, such as mitogen-activated protein kinases (MAPKs) [271,272], protein kinase C (PKC) [271,273,274], AMP-activated protein kinase (AMPK) [275,276,277], PI3K [278,279], and glycogen synthase kinase-3 β (GSK3β) [280], induce the phosphorylation of Nrf2 and participate in Nrf2 transcription. The main regulatory mechanism of Nrf2 stability is, however, maintained through binding with KEAP1 (Figure 3).

**Figure 4:**
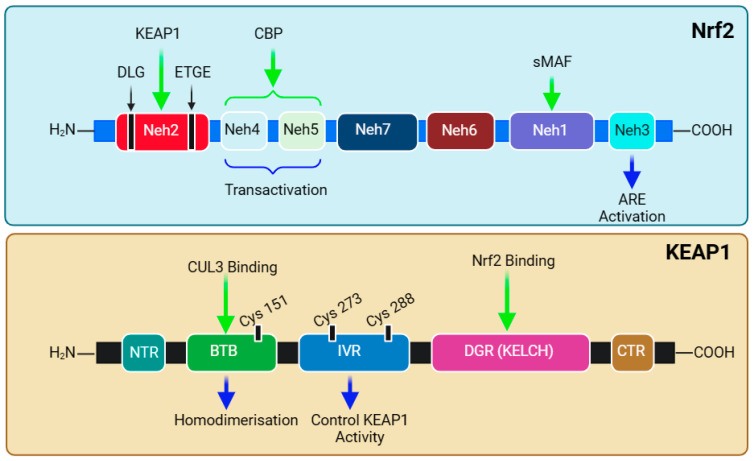
Structural domains of Nrf2 and KEAP1. The protein structure of Nrf2 contains seven domains starting from the amino terminal, Neh2, through Neh4, Neh5, Neh7, Neh6, Neh1, and Neh3, at the carboxyl terminal’s end. The Neh2 domain contains DLG and ETGE as binding motifs for KEAP1. The Neh4 and Neh5 domains are known to be involved in NRF2 transactivation. The Neh1 domain has the DNA-binding motif through heterodimerisation with a small musculoaponeurotic fibrosarcoma (sMAF) protein. KEAP1 has three domains and a region on amino (NTR) and carboxyl (CTR) terminals. The Bric-à-Brac (BTB) domain is responsible for the homodimerisation of KEAP1 and binds to CUL3, and the intervening region (IVR) is involved in the control of KEAP1 activity, while the Kelch domain (KELCH) of DGR binds to the ETGE or DLG motif of the Neh2 domain of NRF2—i.e., one monomer of KEAP1 takes ETGE, while the other binds with DLG. This figure has been generated using Biorender.

The functional adaptation of Nrf2 is based on its seven structural domains, Neh1–7, which attributes its stability and transcriptional activity to target genes (Figure 4). Notably, the *N*-terminal domain (Neh2) is responsible for binding with KEAP1 and, hence, governs the stability and ubiquitination of Nrf2. Studies have shown that high-affinity ETGE (K_d_ ~ 5 nM)- and low-affinity DLG (K_d_ ~ 1 μM)-binding motifs exist and coordinate Nrf2-KEAP1 binding [281,282,283,284]. The Neh2 domain contains seven lysine residues, which are critical substrates in the above-mentioned ubiquitination (binding with ubiquitin) and KEAP1-dependent Nrf2 degradation [269]. The mechanism of attachment and detachment of the Nrf2-KEAP1 in a model called Hinge and Latch mechanism has been described [285,286,287]. The next two domains, Neh4 and Neh5, are involved in interactions with nuclear co-factors and facilitate transcriptional activation. The synergistic cooperation of Neh4 and Neh5 to recruit a coactivator molecule called CBP (cAMP Responsive Element-Binding protein (CREB)-binding protein) has been suggested [288]. The activity of Nrf2 is enhanced by acetylation by CBP [289]. This is followed by the Neh7 domain, which is involved in repressing Nrf2 transcriptional activity via interaction with retinoic X receptor α [290]. Readers should note that Nrf2 was originally discovered in 1994 as a cap’n’collar (CNC) basic-region leucine zipper (bZIP) transcription factor [291]. Through a basic leucine zipper motif, the Neh1 domain is the site for binding Nrf2 to the ARE sequence and is involved in the interaction with the E2 ubiquitin-conjugating enzyme to govern Nrf2 stability and translocation [292]. Hence, the heterodimerisation of Nrf2 with Maf and DNA binding are functions of the Neh1 domain. KEAP1-independent Nrf2 degradation (e.g., by glycogen synthase kinase-3β (GSK-3β) is regulated by the Neh6 domain [293]. At the C-terminal, the Neh3 domain is involved in the interaction with a transcription coactivator called CHD6 (a chromo-ATPase/helicase DNA-binding protein) in the nucleus. Hence, three domains—Neh3, Neh4, and Neh5—are involved in the interaction with the coactivators for transactivation by Nrf2. The structural overview of both Nrf2 and KEAP1 is presented in Figure 4, and their relevance to sulforaphane bioactivity is discussed below.

The cysteine sulfhydryl groups of KEAP1 are the redox sensors regulating Nrf2’s transcriptional activity [294,295,296]. Under non-stressful or normal physiological conditions, the level of Nrf2 is kept low through constant degradation in the cytoplasm. The alteration in the sulfhydryl group of KEAP1 cysteines and the subsequent loss of E3 ligase activity are what that allows the newly synthesised and available Nrf2 to translocate to the nucleus (Figure 3). KEAP1 itself possesses several structural and functional domains (Figure 4), which are not detailed herein. Of note are the domains for the homodimerisation of the KEAP1 broad complex/tramtrack/bric-a-brac (BTB) domain, which binds CUL3 and is involved in KEAP1 homodimerisation [297]. The interaction of KEAP1 with the Neh2 domain of Nrf2 is a function of the so-called Kelch/DGR (double glycine repeat) domain, while the central intervening region (IVR) of KEAP1, which is rich in cysteine residues, controls KEAP1 activity. The DGR and carboxyl-terminal region (CTR) are also called the DC domain. From a functional point of view, forked-stem dimer structures with two large spheres enclosing the intervening double glycine repeat and C-terminal domains have been described by Ogura et al. [298].

The therapeutic implication of Nrf2 in the treatment of inflammatory diseases has been clinically proven. For example, dimethyl fumarate (DMF) has been effectively used in the treatment of relapsing-remitting multiple sclerosis [299,300,301]. The clinical application of DMF in the treatment of psoriasis has also been described [302]. Another potential drug at the development stage is bardoxolone (CDDO-Me), an oleanane-type triterpene acting as an Nrf2 inducer, which has shown promise in the treatment of diabetic nephropathy [303]. Overall, the activation of Nrf2 has been shown to reduce the pathological score of many diseases such as chronic obstructive pulmonary disease (COPD) [304] and sickle cell disease [305], among others. In this context, different therapeutic agents target the various cysteine residues located in KEAP1. Of these, sulforaphane is among the most extensively studied compounds acting as Nrf2 activators both in vitro and in vivo. It targets Cys151 (Figure 3) at the BTB domain of KEAP1 and blocks the KEAP1-CUL3 interaction, thereby reducing Nrf2 ubiquitination [306]. Diethyl maleate (DEM), DMF, and *tert*-butylhydroquinone are other examples of drugs acting in the same way as sulforaphane by preferentially targeting Cys151 [307]. The cyanoenone class of Nrf2 activators has also been shown to be targeting Cys151 in KEAP1, irrespective of their molecular size [308]. Andrographolide has also been shown to partially inhibit the interaction of KEAP1 with CUL3 by targeting Cys151 in KEAP1 [309], while 15-deoxy-Δ^12,14^-prostaglandin J_2_ (15d-PGJ_2_) is one exemplary drug preferentially targeting Cys288, and, in fact, the mutation of Cys151 does not affect Nrf2 induction by this compound [310]. Other agents such as 9-nitro-cotadec-9-enoic (OA-NO_2_) and 4-hydroxynonenal (4-HNE) target Cys151 but also, crucially, Cys273 and Cys288 [311]. In this regard, out of the twenty-seven cysteine residues known to occur in the human KEAP1 protein, the three that are most conserved across many living organisms and that are important targets for drugs are known to be Cys151, Cys273, and Cys288 [310]. A further group of compounds either targeting Cys288 or Cys226, Cys613, Cys622, and Cys624 is also known. Hence, drugs targeting cystine sensors of KEAP1 can be classified into several categories based on the cysteine residues they preferentially target. The readers should also note that not all therapeutic agents of Nrf2 induce work through the KEAP1 pathway. For example, curcumin has been shown to enhance the Nrf2 pathway by activating p38 MAPK to increase the Nrf2 level and the level of Nrf2-ARE interaction [312]. A further note on this subject is also that the upregulation of HO-1 activity and anti-inflammatory effects can be achieved by therapeutic agents independent of the Nrf2 pathway, as shown for some flavonoids [313].

Overall, numerous studies demonstrating the anti-inflammatory effect of sulforaphane have been shown to be associated with the induction of Nrf2 (Table 1, Table 2, Table 3, Table 4 and Table 5). In alveolar macrophages from patients with COPD, the use of siRNA to silence Nrf2 induction completely abolished the effect of sulforaphane upon recognition and the phagocytosis of clinical isolates of nontypeable *Haemophilus influenza* (NTHI) and *Pseudomonas aeruginosa* [65,66]. The induction of TNF-α, IL-1β, and IFN-β production in alveolar macrophages stimulated by LPS could also be suppressed by sulforaphane in the Nrf2-dependent pathway, as revealed by studies using Nrf2 (−/−) mice [71]. Along the same line of evidence, the deletion of KEAP1 in the lungs attenuates acute cigarette smoke-induced oxidative stress and inflammation [314]. The relevance of the induction of Nrf2 for the anti-inflammatory effect of sulforaphane in the various experimental models is clearly shown in Table 1, Table 2, Table 3, Table 4 and Table 5.

### 4.2. Inhibition of NF-κB

**Figure 5:**
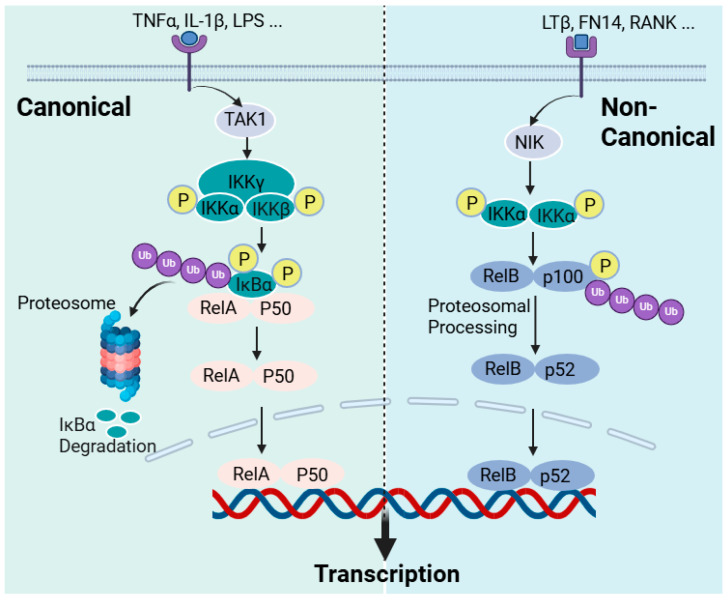
The NF-κB activation pathways. The activation of cellular receptors to pro-inflammatory cytokines or bacterial and viral products leads to the activation of the NF-κB pathways through two principal routes: the canonical and non-canonical pathways. In the canonical NF-κB pathway, receptor activation (e.g., TNF-α, IL-1β, and LPS receptors) triggers the activation of the transforming growth factor beta-activated kinase 1 (TAK1), which activates the inhibitor of kappa B kinase (IKK) via phosphorylation at the IKKβ site. In turn, IKKβ then phosphorylates p105 bound to RelA to initiate proteasomal degradation or processing into p50. IKKβ also phosphorylates IκBα to initiate its proteasomal degradation. The free p50-RelA dimer then translocate to the nucleus to promote target gene activation. In the non-canonical pathway of NF-κB activation, receptor activation such as that of the lymphotoxin β receptor (LTβR), the B-cell-activating factor receptor (BAFF-R), Fn14, the Tweak receptor, and the receptor activator of NF-κB (RANK) leads to NF-κB-inducing kinase (NIK) activation, which phosphorylates the inhibitory kappa B kinase alpha (IKKα). IKKα, in turn, phosphorylates p100 for proteasomal processing into p52. The RelB/p52 dimer, as NF-κB, is then translocated to the nucleus. This figure has been generated using Biorender.

NF-κB is a generic name for a family of dimeric proteins as transcription factors induced by a variety of agents, including pro-inflammatory mediators. These proteins have subunits, five in mammals RelA (p65), RelB, p50 (from p105 precursor), p52 (p100 precursor), and c-Rel, which make dimers, but the most studied so far and the most widely expressed in cells is the p50/RelA dimer, NF-κB. All these subunits are known to have the Rel homology domain (RHD), which is critical for dimerisation, DNA binding, and the interaction with lκB inhibitors. In addition, RelA (also RelB) have transcription activation domains (TADs), which were all well characterised in the 1990s in [315] and the references therein. In unstimulated cells or under normal physiological conditions, the NF-κB dimers are bound to inhibitory molecules of the IκB family of proteins (inhibitors of NF-κB), and the activation of NF-κB requires cleavage from IκB by IκB kinase (IKK). The IKK is an enzyme complex that consists of two kinase subunits, IKKα (IKK1) and IKKβ (IKK2), and a regulatory subunit IKKγ (NEMO). The key to NF-κB activation is the phosphorylation of IκB proteins, inhibitory proteins, on specific serines in the *N*-terminal, leading to ubiquitination and the subsequent proteasomal degradation (Figure 5). Removing the inhibitory proteins from the complex releases NF-κB dimers to translocate to the nucleus and activate target inflammatory genes.

Readers who need to explore the detailed mechanism of the NF-κB activation pathways may refer to review articles on the subject [316,317]. Herein, a brief overview of NF-κB activation that is critically relevant to the discussion on the mechanism of action of sulforaphane is described. NF-κB has a diverse function in the cellular metabolism but is primarily involved in regulating the immune response following pathogenic or stress signals, leading to inflammation. In the innate immune response, immune cells such as macrophages and dendritic cells have receptors for pathogen-associated molecular patterns (PAMPs) that recognise bacteria and viruses. The toll-like receptor (TLR) family, out of which TLR4 is the best-known member, is among these receptors for bacterial products (LPS) that orchestrate the induction of NF-κB. These receptors are collectively called pattern recognition receptors (PRRs). Cell-mediated immunity consists of T lymphocytes-based antigen clearance (through the T-cell receptor (TCR)) and B lymphocyte (through the B-cell receptor) humoral immune responses, which also require NF-κB activation. In this classic example of immune cells’ activation by bacteria or their products, NF-κB is activated to initiate the inflammatory response, which includes the expression of pro-inflammatory cytokines (TNF-α, IL-1, and IL-6), chemokines, adhesion molecules, etc. This is what is called the “canonical” pathway of inflammation and can also be induced by pro-inflammatory cytokines such as TNF-α and IL-1, with the NF-κB involved being RelA- or cRel-containing complexes (Figure 5). Hence, the activation of TNF receptors, TLRs, T-cell receptors, and interleukin receptors typically leads to the activation of the canonical pathway of inflammation. Various inflammatory diseases, including rheumatoid arthritis, inflammatory bowel disease, asthma, and chronic obstructive pulmonary disease, are products of the exaggerated inflammatory response via the canonical NF-κB pathway. An “alternative” or “non-canonical” NF-κB activation pathway leading to the activation of RelB/p52 complexes can also occur by induction via lymphotoxin β, the B-cell-activating factor, and the receptor activator of the NF-κB ligand, among others. As indicated above, IKKα and IKKβ (catalytic units) and IKKγ (regulatory subunit) constitute the classical canonical NF-κB activation mechanism. IKKβ regulates the activation of the canonical pathway through the phosphorylation of IκBs and requires the IKKγ subunit, while IKKα is required for the activation of the alternative pathway (Figure 5). IKK itself is activated in the canonical pathway via receptor activation through phosphorylation by kinases and, most notably, the TGFβ-activated kinase 1 (TAK1; also known as MAP3K7), but there are many others, including RIP1 (receptor-interacting kinase 1) and TBK1 (TANK-binding kinase) [318]. The phosphorylation of IKKβ is also known to be involved in the processing of p105 to yield p50 [319]. Hence, the canonical activation of NF-κB is regulated through phosphorylation by IKKβ for the proteasomal degradation of IκBα, leading to RelA-p50 complexes’ translocation to the nucleus. On the other hand, the non-canonical pathway of NF-κB activation is the process of releasing RelB-p52 dimers through the phosphorylation of IκB, and, for this, IKK phosphorylation is a function of the NF-κB-inducing kinase (NIK; also known as MAP3K14). The link between NIK phosphorylation and the activation of TNF superfamily receptors (TNFSFRs) such as TNFSFR12A (Fn14, Tweak receptor), the lymphotoxin β receptor (LTβR), the B-cell-activating factor receptor (BAFF-R), the receptor activator of NF-κB (RANK), CD40, and CD27 has been established [320,321]. Further details are available from review articles on the subject [316,317]. The regulatory mechanism of NF-κB in the B-cell survival and maturation, dendritic cell activation, bone metabolism, and lymphoid organogenesis is known to involve the alternative or non-canonical pathway [322,323]. In terms of therapeutic intervention, the NF-κB pathway has been extensively studied in the last few decades as a potential target for anti-inflammatory compounds. By far, the most known specific target of this for natural products is *IKK*, predominantly targeting IKKβ. The diverse function of IKK, however, remains a challenge for the therapeutic application of these agents and, specifically, for targeting inflammatory diseases. Research on this field is extensively diverse, and the inhibitory compounds belong to diverse structural groups, including natural products such as terpenoids, flavonoids, or polyphenols, among others. Thiol-reactive compounds such as sulforaphane are included in this group [324].

On the above basis, sulforaphane has been shown to downregulate NF-κB activity in the following cell types under a variety of inflammatory conditions: bone marrow-derived dendritic cells [74]; T-cell lines [81]; PBMCs [82]; THP-1 [86,100]; RAW264.7 cells [94,95,97]; human mast cells [102]; mouse microglial BV2 cells [103,113,114]; N9 murine microglial cells [105]; primary rat microglia [114]; the C6 astrocyte cell line [116]; neuroblastoma SH-SY5Y cells [161]; N2a/APPswe cells [162]; HUVECs [121,122,126]; ECV304 [124,125]; human saphenous vein endothelial cells [127]; primary goat mammary epithelial cells [130]; human retinal pigment epithelial [134]; human mammary epithelial [140]; VSMCs [149,150]; primary human articular chondrocytes [153]; retinal pigment epithelial [154]; synoviocytes [155]; human embryonic kidney 293T (HEK293T) cells [156]; and mouse pancreatic acinar cells [158].

Mechanistically, sulforaphane, by virtue of forming dithiocarbamate through a reaction with thiol groups, can directly interact with DNA binding and the transactivation of NF-κB [325]. It has also been shown to suppress TNF-α-induced IκBα phosphorylation and IκBα degradation [326]. In TNF-α-stimulated HUVECs and human aortic endothelial cells, sulforaphane has inhibited the induced phosphorylation of IκB kinase (IKK) and IκB-α, as well as the binding of NF-κB to binding sites in the LIPG gene [126]. Through direct interaction with cysteines, sulforaphane also inhibits the IκBβ subunit and IκBα [95] and the phosphorylation of IκBα [102,126,130]. It is also reported to inhibit transcriptional activity as well as IκBα phosphorylation and degradation [121,122,124,140,150]. Evidence of the inhibition of the DNA-binding activity of NF-κB [153] as well as C/EBP and CREB binding [94] or the binding of NF-κB to binding sites in the LIPG gene, has also been shown [126]. Collectively, there is overwhelming evidence to suggest that the anti-inflammatory effect of sulforaphane is mediated through the downregulation of NF-κB at the various stages of its signalling cascade. The crosstalk between NF-κB activation and signalling through tyrosine kinases is discussed below (Section 4.4).

### 4.3. Nrf2-NF-κB Crosstalk

**Figure 6:**
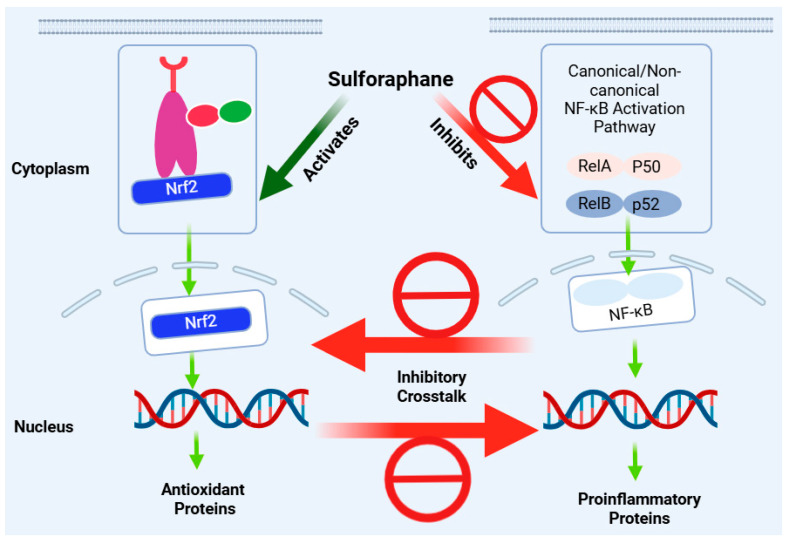
Anti-inflammatory effect of sulforaphane via crosstalk of the Nrf2 and NF-κB pathways. Inhibition of the NF-κB pro-inflammatory pathway is mostly associated with the induction of the Nrf2 pathway as an antioxidant and anti-inflammatory mechanism. Both effects are simultaneously seen for sulforaphane in various experimental models, although the magnitude of modulation and interdependence of the two pathways may differ. This figure has been generated using Biorender.

In the regulation of redox status and inflammation, there is a great deal of coordination between the Nrf2 and NF-κB pathways. Numerous reports have shown that stress conditions that activate the NF-κB pathway, such as that of TNF-α or pathologies such as cancer, also stimulate Nrf2’s protective mechanisms [327,328]. Generally, the activation of NF-κB can lead to the upregulation of Nrf2 or its target gene products, such as HO-1. In many respects, however, the inflammatory response mediated through the NF-κB pathway is counteracted by Nrf2 activation [329,330] (Figure 6). For example, the degradation of IKKβ by KEAP1, leading to decreased phosphorylation and the negative regulation of the NF-κB pathway, has been described [331]. The competition between Nrf2 and the NF-κB-p65 subunit to bind the transcriptional coactivator CBP has also been demonstrated [332]. The Nrf2 pathway has also been shown to suppress the transcription of several inflammatory cytokine genes [333]. In this regard, many anti-inflammatory agents that suppress NF-κB signalling have been shown to activate the Nrf2 pathway. These include α-tocopheryl [334].

In peritoneal macrophages, sulforaphane has been shown to inhibit the mRNA expression of TNF-α, IL-1β, COX-2, and iNOS induced by LPS, but this effect is evident in (+/+) but not in Nrf2 (−/−) peritoneal macrophages [71]. In almost all cases where both Nrf2 and NF-κB activation were studied, the effect of sulforaphane was associated with enhancing Nrf2 while, at the same time, downregulating NF-κB (Table 1, Table 2, Table 3, Table 4 and Table 5). Unequivocal evidence of crosstalk comes from knockdown studies where the effect of sulforaphane has been reported to be dependent on Nrf2 activation: for example, the inhibition of the NLRP3 inflammasome in an Nrf2-independent manner [98]. This could be a partial effect, not a complete one, as the knockdown of Nrf2 has been shown to partly (not completely) abolish the reduction in ROS, nitric oxide, and pro-inflammatory cytokines (TNF-α, IL-1β, and IL-6) [105]. Hence, multiple mechanisms of action, often paradoxical, may be at play in the anti-inflammatory effect of sulforaphane.

### 4.4. Signalling Paradox

In macrophages, the inhibitory effect of sulforaphane on cytokine expression and M1 markers’ C-C motif chemokine receptor 7, IL-23, and iNOS has been shown to be associated with the inhibition of mitogen-activated protein kinases (MAPK), p38, c-Jun N-terminal kinase (JNK), and phosphorylation [99]. Downregulating the phosphorylation of MAPK in PMA-activated human mast cells using sulforaphane has also been associated with the inhibition of NF-κB and cytokine expression [102]. PMA-activated endothelial cells (HUVECs) have also been shown to respond to sulforaphane treatment by reducing the level of pro-inflammatory cytokine (IL-1β and TNF-α) expression via the downregulation of the phosphorylation of p38, extracellular regulated kinases (ERK) 1/2, and JNK [123]. Another example of PMA-activated NF-κB activation can be found in the human mammary epithelial cells, where sulforaphane inhibits both IKK activation and ERK1/2—i.e., ERK1/2-IKKα-NF-κB signalling [140]. In ECV304 endothelial cells, the suppressive effect of sulforaphane on NF-κB translocation has been shown to be mediated via the inhibition of the phosphorylation of mainly p38 MAPK and JNK MAPK [125]. The expression of adhesion molecules on endothelial cell surfaces induced by TNF-α has also been shown to be inhibited by sulforaphane via the inhibition of the activation of p38 MAPK (not JNK), and, interestingly, this effect is not mediated via Nrf2 expression [129]. The phosphorylation levels of ERK and JNK MAPKs associated with cigarette smoke extract (CSE)- and particulate matter-induced inflammatory and chemokine gene (IL-1β, IL-6, IL-8, TNF-α, MCP-1, and CXCL-1) expression in human lung epithelial cells are further decreased by sulforaphane [136]. In BV2 microglial cells treated with advanced glycation end products, the expression of neuroinflammatory mediators and ROS is inhibited by sulforaphane in association with reduced levels of GSK3β activation and p38 phosphorylation (but not ERK and JNK phosphorylation) and the inhibition of NF-κB. All these data suggest that the anti-inflammatory effect of sulforaphane via suppressing the NF-κB activation pathway is associated with the suppression of the MAPK pathway and is not necessarily associated with Nrf2 activation. On the other hand, the induction of Nrf2 in microglial cells has been shown to be dependent on the phosphorylation of p38 and ERK1/2, and sulforaphane augments this activity [105]. It is also known that cigarette smoke generally activates MAPK signalling cascades in lung epithelial cells both in vitro and in vivo [335]. In this regard, the inhibition of p38 MAPK is proven to ameliorate allergen-induced pulmonary eosinophilia, mucus hypersecretion, and airway hyper-responsiveness or diseases such as asthma and COPD. While Nrf2 is experimentally proven to have a protective role in numerous airway diseases [336], the signalling pathway in collaboration or contradiction with NF-κB is still not clear. In a study using human lung epithelial cells, where inflammatory cytokines were inhibited by sulforaphane, MAPKs were inhibited while Nrf2 was activated [136]. Overall, sulforaphane seems to block the phosphorylation of MAPKs (p38, JNK, and ERK1/2), along with NF-κB p65 [113].

Studies have shown that the upregulation of phosphoinositide 3-kinase (PI3K) and Akt by therapeutic agents (e.g., ginkgolides and bilobalide) can activate Nrf2 [337]—i.e., Akt, as a downstream signal molecule of PI3K, can phosphorylate KEAP1 as well as have other effects, leading to Nrf2 activation. In this context the hypoxia- or cobalt chloride-induced upregulation of TLR4 mRNA and protein have been shown to be mediated by inhibiting PI3K/Akt activation, and sulforaphane reverses this effect to induce protection against oxidative stress in macrophages [88]. PI3K activation inhibits macrophage programming into M1, while Akt activation is a critical condition for M2 polarisation. Thus, sulforaphane suppresses the cobalt chloride-induced upregulation of TLR4 by inhibiting PI3K/Akt activation and the subsequent nuclear accumulation and transcriptional activation of HIF-1α [88].

### 4.5. Anti-Inflammatory Effect of Sulforaphane by Targeting Sirtuin 1 (SIRT1) Signalling

Sirtuin 1 (SIRT1) belongs to a family of proteins (SIRT1-7; silent information regulators or sirtuins) or deacetylase enzymes that function in collaboration with their essential co-factor, nicotinamide adenine dinucleotide (NAD^+^). They remove acetyl groups from the lysine residues of many proteins or histone substrates and function in the post-translational modification of proteins by mono-ADP ribosylation. As shown in the section below, SIRT1 also catalyses a range of substrates such as NF-κB, forkhead box class O family member proteins (FOXOs), peroxisome proliferator-activated receptor γ (PPARγ), peroxisome proliferator-activated receptor γ coactivator 1α (PGC-1α), and P53 [338,339,340]. In terms of inflammation, NF-κB and SIRT1 display antagonistic crosstalk, where NF-κB is acting in a pro-inflammatory manner while SIRT1 promotes anti-inflammatory responses. While SIRT1 overexpression reverses the inflammatory pathology, its underexpression promotes inflammation, as shown in experimental animals exposed to cigarette smoke or chronic obstructive pulmonary disease [341,342]. In macrophages, the knockdown of SIRT1 has been shown to increase the activation levels of NF-κB and pro-inflammatory cytokines [343]. These experiments have revealed that SIRT1 overexpression decreases the acetylation of RelA/p65 and NF-κB-dependent inflammation and/or NF-κB activity by inducing suppressor mechanisms. Hence, the upregulation of SIRT1 reduces COX-2 levels by inhibiting the activation of AP-1 and NF-κB [344]. Overall, SIRT1 activation could exert benefits in the treatment of inflammatory disorders, and its level/activity is suppressed by NF-κB through the modulation of downstream signalling. For example, through NF-κB induction, TNF-α inhibits the expression of peroxisome proliferator-activated receptor gamma coactivator-*1*α (PGC-1α), which plays critical role in SIRT1 signalling. Accordingly, SIRT1 activation suppresses the expression of TNF-α in macrophages [345]. Another important signalling molecule related to SIRT1 is the AMP-activated protein kinase (AMPK), which acts as an initial sensor to increase the level of NAD^+^ [346].

**Figure 7:**
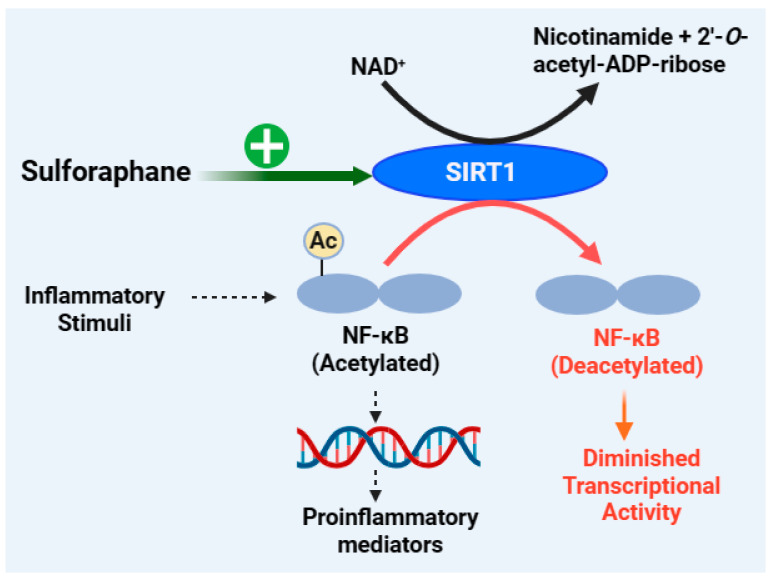
Anti-inflammatory effect of sulforaphane through the SIRT1 mechanism. The normal function of NF-κB in the induction of inflammatory gene/protein (e.g., IL-1, IL-6, TNF, ICAM, VCAM, ELAM, iNOS, COX-2, etc.) expression requires the acetylation process. By deacetylating NF-κB, SIRT1 acts as a negative regulator of inflammation or NF-κB—i.e., SIRT1 denies NF-κB its DNA binding, transcriptional activity, and stability as well as interaction with protein modifiers. Boosting the expression of SIRT1 with sulforaphane thus reduces the NF-κB-dependent inflammatory response as well as other effects associated with the deacetylation activity of SIRT1 and several other inflammatory proteins. This figure has been generated using Biorender.

The inhibition of NF-κB p65 nuclear translocation by sulforaphane in human retinal pigment epithelial (ARPE-19) cells exposed to blue light is coupled with the Nrf2 pathway, which, in turn, is associated with increased protein expression of SIRT1 and PGC-1α gene expression [134]. Sulforaphane also upregulates the phosphorylated level of AMPK (p-AMPK), SIRT1, and PGC-1α to ameliorate LPS-induced changes in intestinal permeability, oxidative stress, inflammation, and apoptosis [135]. The upregulation of p-AMPK, SIRT1, and PGC-1α has also been observed in LPS-stimulated Caco-2 cells after treatment with sulforaphane, which is also coupled with the upregulation of antioxidant enzymes (e.g., SOD, GPx, and CAT) and the downregulation of inflammatory cytokines (IL-1β, IL-6, IL-8, and TNF-α) [135]. Oligomeric amyloid-β_1-42_ can trigger injury in the retinal pigment epithelium by repressing SIRT1, but treatment with sulforaphane can maintain cell viability and SIRT1 expression [347]. Sulforaphane prevents rat cardiomyocytes from hypoxia/reoxygenation injury in vitro by elevating the expression of SIRT1 in cardiomyocytes [348]. It also exerts an anti-apoptotic effect on chondrocytes and ameliorates the OA in vivo by activating SIRT1 [166]. In support of this mechanism, many natural products, including resveratrol as an allosteric activator of SIRT1 [349] and curcumin [350], have been shown to induce cardioprotective effects by activating SIRT1. Hence, SIRT1 activation is an emerging mechanism of action for the anti-inflammatory effect of sulforaphane, as depicted in Figure 7.

### 4.6. Anti-inflammatory Effect of Sulforaphane by Targeting STATs

The roles of the silent information regulator sirtuin 1 (STAT1) and STAT3, often as contradictory as pro- and anti-inflammatory mediators in macrophages, have been widely reported [351]. Thus, LPS and IL-6 can activate the M1 phenotype, and IL-10 acts as an anti-inflammatory cytokine through the modulation of STAT1 and STAT3 signalling [352]. STAT3 activation is generally considered to promote the anti-inflammatory M2 phenotype, in contrast to NF-κB activation, which promotes the M1 phenotype associated with the expression of pro-inflammatory cytokines. Thus, therapeutic agents that reduce the activation of STAT1 and/or suppress STAT3 may induce an anti-inflammatory effect by promoting the polarisation of M1 to the M2 phenotype [353]. For example, Sun et al. [73] have reported that sulforaphane enhances IL-10 production in macrophages while promoting STAT3 activation. On the other hand, the suppressive effect of sulforaphane on LPS-mediated increase in ICAM-1 and VCAM-1 expression on the endothelial (HUVEC) cell surface has been unequivocally proven (siMRA study) to be associated with the inhibition of STAT3 [120]. This finding agrees with that reported by Rakariyatham et al. [97] on macrophages stimulated by LPS, where the level of STAT3 phosphorylation was inhibited by sulforaphane. Furthermore, Jeong et al. [160] have shown that TNF-α-induced NF-κB as well as STAT1 activation in human keratinocytes is inhibited by sulforaphane. Hence, further research is needed to establish the cell- and/or inflammatory model-dependent role of STAT1/3 as a target for sulforaphane.

### 4.7. The Emerging Role of Activator Protein-1 (AP-1) as an Anti-Inflammatory Target for Sulforaphane

Just like NF-κB, activator protein-1 (AP-1) represent a family of transcription factors functioning as dimers to regulate immune function and oncogenic processes. Of note, it is linked to MAPK signalling and is involved in T-cell activation. AP-1 proteins play important roles in the development and maintenance of cancers, which are not described herein. Their role as a therapeutic target for inflammatory diseases has also been established [354] and includes chronic inflammatory diseases such as rheumatoid arthritis [355]. This is attributed to AP-1 being pro-inflammatory, as NF-κB, and able to regulate the expression of cytokines such as TNF-α and IL-1 through direct interaction with their promoter AP-1-binding motifs. As it has been shown for sulforaphane, the crosstalk between redox and inflammatory regulation for AP-1 follows the same line of evidence as NF-κB. Hence, the suppressive effect of sulforaphane on LPS-induced COX-2 expression has been shown to be associated with the inhibition of NF-κB, C/EBP, and CREB, as well as AP-1 [94]. By reducing the level of JNK phosphorylation, sulforaphane has been demonstrated to suppress NF-κB and AP-1 signalling in LPS-activated microglia [115]. The photoprotective effects of sulforaphane in human keratinocyte cells and BALB/c mice subjected to repetitive ultraviolet A (UVA) irradiation have been shown to be associated with the inhibition of MAPK/AP-1 signalling [356]. In a similar study, using UVB-induced AP-1 activation, sulforaphane directly inhibited the DNA-binding activity of AP-1 [357]. Further studies have shown that the inhibition of AP-1 by sulforaphane involves interaction with cysteine in the cFos DNA-binding domain [358]. Evidence is also rich on the anticancer effect of sulforaphane by targeting AP-1. In prostate cancer cells, for example, AP-1 activation is attenuated by the combinations of sulforaphane and epigallocatechin gallate [359]. Other known anti-inflammatory compounds of natural origin that have shown an effect through the AP-1-dependent pathway include quercetin [360], omega-3 docosahexaenoic fatty acid [361], ganglioside GM3 [362], and curcumol [363].

As with NF-κB, paradoxical evidence of AP-1’s role as a target for sulforaphane has been noted. For example, the treatment of breast cancer cells with sulforaphane inhibits the TPA-stimulated NF-κB-binding activity but not the AP-1-binding activity [364]. Hence, more research is needed, although, overall, AP-1 inhibition, just like NF-κB, appears to be a common mechanism for sulforaphane as an anti-inflammatory compound.

### 4.8. MicroRNAs as Potential Therapeutic Targets for Sulforaphane

MicroRNAs (miRNAs) are small-molecular-weight non-coding RNAs that suppress gene expression both by inhibiting protein translation and promoting mRNA cleavage. In principle, they can either enhance or inhibit inflammation, depending on the targeted mRNAs. As reviewed by Tahamtan et al. [365], several miRNAs are known to act as anti-inflammatory agents, and they include miR-10a, miR-21, miR-24, miR-106b, miR-124, miR-143, miR-145, miR-146, miR-155, and miR-375. They can act on several target transcription factors such as NF-κB and STATs, receptors such as TLR, TNF receptors, or even kinase enzymes. In the LPS-stimulated RAW264.7 cells, sulforaphane has been shown to suppress the miR-146a and miR-155 levels associated with inflammation [90,91]. The enhanced miR-423-5p levels associated with hepatic stellate cell activation in liver fibrosis have also been shown to be downregulated by sulforaphane [366]. In contradiction to such an effect, the level of miR-423-5p has been shown to be low in septic mice with a liver injury, and its overexpression alleviates acute liver injury and inflammatory responses [367]. Hence, more research in the miRNA field is needed to establish the contribution of miRNAs as targets for sulforaphane as an anti-inflammatory agent.

## 5. General Summary and Conclusions

The present analysis of sulforaphane as an anti-inflammatory agent clearly shows the crosstalk between inflammation and oxidative stress, specifically via NF-κB and Nrf2 signalling. Sulforaphane appears to suppress both inflammation and oxidative stress mostly by inhibiting NF-κB and enhancing Nrf2 signalling. The long list of publications based on sulforaphane suppressing inflammation in vivo and in vitro has been described in the previous sections through the effects attributed to the modulation of NF-κB and Nrf2. However, there have been paradoxical results against this conclusion, and more research is needed to clarify the degree of interdependence between the two pathways in mediating the anti-inflammatory effect of sulforaphane. The readers should also note that there are also some studies suggesting that sulforaphane could have pro- and anti-inflammatory effects, as revealed for placental cytokine production under some conditions of bacterial infections [368]. Furthermore, there are also studies that show that sulforaphane improves the oxidative status under ischaemia/reperfusion injury in rats without attenuating the inflammatory response [369]. Indeed, in some studies, the pro-oxidant nature of sulforaphane is well known and, with it, one of the mechanisms for its anticancer effect. As a pro-oxidant compound, sulforaphane synergises with anticancer agents such as cisplatin to induce apoptosis through ROS accumulation [370]. This paradoxical effect could be dose-dependent, as most of the anti-inflammatory effects in vitro are carried out at 5 or 10 µM, while higher doses are known to increase ROS and even inhibit ROS-scavenging enzymes such as GR and GPx activity in cancer cells [371]. By interfering with the glutathione recycling processes, sulforaphane can also induce oxidative stress and death through the p53-independent mechanism [371]. In the cancer domain, sulforaphane also augments the immune response instead of having an immunosuppressive effect, as shown in the WEHI-3-induced leukaemia mouse model, where it enhances phagocytosis by macrophages and natural killer cells [372]. Along the same line, Nrf2 activation by sulforaphane restores the age-related decrease in TH1 immunity by acting on dendritic cells, implying that it acts as an immunostimulant [373]. While it induces a cytoprotective effect via the Nrf2 mechanism in naïve cells, it actually promotes apoptosis in TNF-α-stimulated synoviocytes [155]. Hence, the effects of sulforaphane as an anti-inflammatory and cytoprotective agent depend on a variety of factors, including the health and disease states. Under hepatitis B virus (HBV) infection in vitro and in vivo, sulforaphane promotes macrophages to the pro-inflammatory M1 phenotype [374], while, under inflammatory conditions both in vitro and in vivo, it induces macrophages to change to the anti-inflammatory M2 phenotype [375], as explained in the various sections of this article.

**Figure 8:**
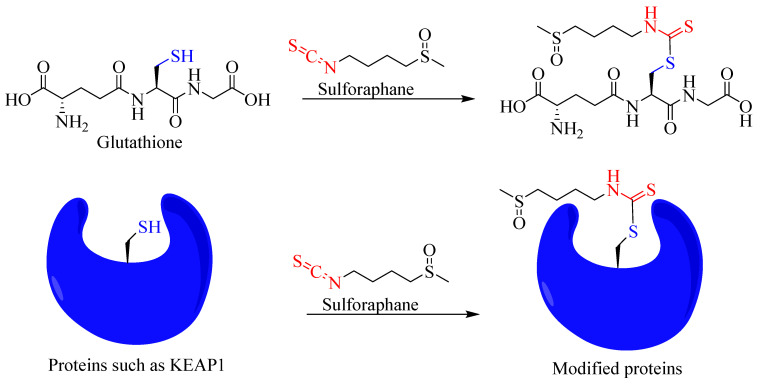
Overview of covalent interaction of sulforaphane with the sulfhydryl group of biomolecules.

As readers can see from the extensive literature cited in this article in the context of inflammation as a disease target, sulforaphane is among the most widely studied natural products both in vitro and in vivo. Mechanistically, its paradoxical effects depending on health and pathophysiological states need further research. Most of the studies show that its effect via interactions with biological molecules such as the cysteine residue of proteins account to its diverse pharmacology. From small molecules such as glutathione to enzymes and transcriptional activities, sulforaphane has been shown to covalently interact with the -SH functional group to induce its diverse functions (Figure 8).

**Figure 9:**
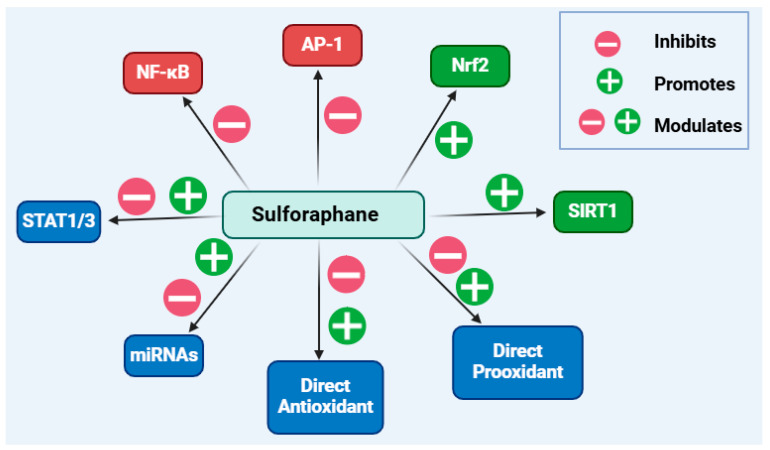
Overview of the anti-inflammatory mechanisms of sulforaphane. Boosting the level and functional activity of Nrf2-dependent genes/proteins while inhibiting the pro-inflammatory mechanisms induced by the transcription factors NF-κB and AP-1 are its main anti-inflammatory mechanisms. Direct interaction of sulforaphane with biological molecules as a pro-oxidant or antioxidant compound may attribute to its biological actions. Evidence also suggests modulatory effects on STAT1 or STAT3 as well as some miRNAs, although further research is needed to clarify the detailed mechanisms of these effects. This figure has been generated using Biorender.

Overall, this article has shown the multiple targets for sulforaphane as an anti-inflammatory compound, which can be summarised as shown in Figure 9. On the basis of the available data, further research on leading optimisation and clinical studies on sulforaphane as an anti-inflammatory agent is well merited.

## Figures and Tables

**Table 1:** Anti-inflammatory effect of sulforaphane via the modulation of leucocyte function.

Cellular Model and Treatment	Concentration	Key Findings	Reference
Neutrophils and PBMCs from healthy volunteers	140 or 280 µM (note the high dose used)	Treatment reduces ROS production, the release of myeloperoxidase from azurophilic granules, and inflammatory cytokines (TNF-α and IL-6) and suppresses phagocytosis.	Wakasugi-Onogi et al. [64]
Alveolar macrophages from patients with COPD	10 µM	Activation of Nrf2 restored bacteria recognition and phagocytosis of clinical isolates of nontypeable *Haemophilus influenza* and *Pseudomonas aeruginosa*; Nrf2-dependent effect confirmed by siRNA.	Harvey et al. [65,66]
Alveolar macrophages from patients with COPD	5 μM	Glutathione-dependent effect activates Nrf2 to *HDAC2* and restores dexamethasone sensitivity.	Malhotra et al. [67]
Alveolar macrophages from alcohol-fed rats	5 μM	Treatment reverses the decrease in cellular RAGE expression and phagocytosis power—effect similar with a glutathione supplement.	Staitieh et al. [68]
Alveolar macrophages from HIV-1 transgenic rats; rat macrophage cell line (NR8383 cells) treated with the HIV-related proteins gp120 or Tat; human monocyte (from peripheral blood)-derived macrophages infected with HIV-1	5 μM	Treatment reverses the decrease in protein expression of Nrf2, NQO1, and GCLC and improves their phagocytic function (confirmed by siRNA to Nrf2).	Staitieh et al. [69]
Porcine pulmonary alveolar macrophages stimulated by LPS	5 µM	Treatment suppresses TRAM, TRIF, RIPK1, TRAF3, TNF-α, IL-1β and IFN-β, and DNMT3a expression. Effect mediated via the suppression of CD14 activation.	Yang et al. [70]
LPS-stimulated peritoneal macrophage from Nrf2 (+/+) and Nrf2 (−/−) mice	5, 10, or 20 µM	Treatment suppresses induced mRNA expression, protein expression, and production of TNF-α, IL-1β, COX-2, and iNOS and HO-1 expression in Nrf2 (+/+) but not in Nrf2 (−/−) macrophages.	Lin et al. [71]
LPS-simulated murine bone marrow-derived macrophages	5 or 10 µM	Treatment diminishes M1 marker expression (IL-1β, IL-6, TNF-α, iNOS, NO, and ROS).	Bahiraii et al. [72]
LPS plus IFN-γ-stimulated bone marrow-derived macrophage from mice	10 μM	Treatment decreases the levels of IL-1β, TNF-α, and IL-6, induces M1-to-M2 phenotype polarisation (cell marker analysis), and promotes STAT3 activation and the production of IL-10.	Sun et al. [73]
Bone marrow-derived dendritic cells co-cultured with CD4+ T-cells isolated from the spleen and lymph nodes of mice activated by anti-CD3ε and anti-CD28 Abs stimulated by LPS	0.1 μM	Treatment inhibits TLR4-induced IL-12 and IL-23 production, suppresses Th1 and Th17 development of T-cells, increases HO-1 expression, and inhibits NF-κB p65 activity.	Geisel et al. [74]
LPS-stimulated PBMC and adipose tissue macrophages	40 µM	Treatment reduces TNF-α, IL-1β, and inflammasome gene expression.	Williams et al. [75]
Human monocyte-derived dendritic cells	10 µM	Treatment reduces the expression of cell-surface markers (CD80, CD83, CD86, HLA-DR, and PD-L1) and Th2 proliferative response, with a decrease in the IL-9 and IL-13 levels, and increases IL-10 levels.	Fernandez-Prades et al. [76]
LPS-stimulated human PBMC- or THP-1-derived macrophages	25 μM	Treatment reduces the expression levels of M1 marker genes, upregulates the M2 marker gene MRC1, decreases the intracellular *S. aureus* load while increasing the intracellular survival of *E. coli* in THP-1 but not in PBMC, and suppresses IL-1β, IL-6, and TNF-α gene expression.	Ali et al. [77]
Human peripheral blood mononuclear cells stimulated by acrolein	1, 5, or 10 µM	Treatment suppresses ROS generation by upregulating Nrf-2 expression and suppresses COX-2 and PGE2 levels.	Qin et al. [78]
Human peripheral blood mononuclear cells stimulated with an anti-CD3 monoclonal antibody	1, 5, or 10 μM	Treatment inhibits the production of IL-6, TNF-α, and IL-17.	Moon et al. [79]
PBMC stimulated by LPS and viral (imiquimod) TLRs	10 or 50 µM	Treatment reduces the pro-inflammatory cytokines (IL-6, IL-1β, and MCP-1) irrespective of TLR stimulations and reduces the proportion of NK cells and monocytes while increasing the proportion of DCs, T-cells, and B-cells.	Mazarakis et al. [80]
Monocytes and CD4+ T-cells infection by HIV (monocyte (THP89GFP and U1) as well as T-cell lines (J89GFP and ACH-2))	10 μM	Treatment suppresses the reactivation of HIV-1 and antagonises the reactivating agents (TNF-α and PMA)—an effect dependent on Nrf2 activation and the downregulation of NF-κB.	Jamal et al. [81]
PBMCs and monocytes from the blood of children with autism spectrum disorder	5 μM	Treatment reverses the deficiency in Nrf2 release, reduces SOD1, GPx1, and GR, and suppresses NF-κB signalling, pro-inflammatory (IL-1β, iNOS, and IL-6) proteins, and mRNA expression stimulated by LPS.	Nadeem et al. [82]
Monocyte-derived macrophages from patients with COPD—LPS- or Pam3CysSerLys4 (Pam3CSK4)-induced inflammation	20 μM	These cells have high levels of TLR2, TLR4, and downstream MyD88 expression, as well as IL-6 and TNF-α levels, compared to normal cells. Their activation further increases these levels, which was supressed by sulforaphane.	Zeng et al. [83,84]
THP-1 or PBMC differentiated by PMA and treated with LPS and IFNγ	10 μM	Treatment shifts macrophage polarisation to a direction specific to the M2 phenotype (CD36 high and CD197 extremely low); this effect was associated with the inhibition of COX-2 expression via the stimulation of MEK-1/2 and JNK1/2 (partial inhibition) to reduce COX-2 expression, but not in p38.	Pal et al. [85]
Human monocytic THP-1 treated with mycoplasma-derived membrane lipoprotein or its analogue, MALP-2	0.5, 1, or 5 μM	Treatment upregulates Nrf2 and HO-1 expression and inhibits TNF-α, IL-1β, and IL-8 secretion and NF-κB activation; a selective inhibitor (SnPP) of HO-1 reversed the inhibitory actions, while a carbon monoxide-releasing molecule (CORM-2) caused a significant decrease in MALP-2-induced cytokine secretion.	Luo et al. [86]
LPS-stimulated J774.1 or RAW264.7 macrophage	5 μM	Treatment activates Nrf2, leading ferroportin 1 (iron exporter) expression and iron release, which reverses the effect of LPS on iron sequestration via the downregulation of ferroportin 1 expression.	Harada et al. [87]
RAW264.7 cells exposed to hypoxia (<1% O_2_) or cobalt chloride (CoCl_2_)	10 or 20 µM	Treatment suppresses the induced upregulation of the TLR4 mRNA and protein by inhibiting PI3K/Akt activation and the subsequent nuclear accumulation and transcriptional activation of HIF-1α (confirmed by selective inhibitor and siRNA knockdown studies).	Kim et al. [88]
LPS-stimulated RAW 264.7 cells	2.5 or 5 µM	Treatment suppresses iNOS and COX-2 expression and inhibits TNF-α, IL-1β, and IL-6 production.	Ranaweera et al. [89]
LPS/IFN-γ-stimulated RAW264.7 cells	10 or 20 µM	Treatment suppresses *iNOS* gene expression and the production of NO, IL-6, TNF-α, and IL-1β via activating the gene expression (mRNA expression) of Nrf2 and HO-1.	Ruhee et al. [90]
LPS/IFN-γ-stimulated RAW264.7 cells	10 or 20 µM	Treatment inhibits the induction of iNOS, TNF-α, and IL-6 and attenuates miR-146a and miR-155 levels.	Saleh et al. [91]
LPS-stimulated RAW264.7 cells	5, 10, or 20 µM	Treatment suppresses TNF-α, IL-6, and iNOS (mRNA and protein) levels, suppresses miR-146a and miR-155 levels, and attenuates the further increase in these inflammation markers by doxorubicin.	Sato et al. [92]
LPS-stimulated RAW264.7 cells	5, 10, or 20 µM	Treatment suppresses NO, iNOS, COX-2, and IL-1β production, inhibits ROS level while enhancing CAT, GPx, Nrf2, NQO1, and HO-1, and, in combination with acetaminophen, increases activity.	Vuong et al. [93]
LPS-activated RAW264.7 cells	15 µM	Treatment suppresses COX-2 protein and mRNA expression, inhibits NF-κB activation but not IκB degradation, inhibits C/EBP- and CREB-binding activity, and inhibits JNK phosphorylation.	Woo et al. [94]
LPS-stimulated RAW 264.7 cells and human monocytes isolated from blood	2-20 µM	Treatment suppresses the expression and release of pro-inflammatory mediators (IL-1β, IL-6, TNF-α, and MMP-9), inhibits antibody-independent phagocytic and chemotactic migratory abilities, suppresses NF-κB and MAPK (p38 and JNK) signalling, and interacts with the cysteines in IKKβ—IκBα.	Reddy et al. [95]
LPS-activated RAW264.7 cells	0.3 or 0.6 μM	Treatment decreases iNOS and COX-2 protein expression levels, induces HO-1 protein expression, and suppresses 0IL-1 and TNF-α mRNA levels, a synergistic effect with nobiletin.	Guo et al. [96]
LPS-stimulated RAW264.7 macrophages	1 μM	Treatment inhibits NO production, reduces the expression levels of pro-inflammatory proteins involving the NF-κB pathway, as well as STAT3 activation, suppresses inflammatory proteins such as iNOS, COX-2, IL-6, and IL-1β, reduces the ROS level in cells, and increases the expression of Nrf2 and HO-1, a synergistic effect with luteolin.	Rakariyatham et al. [97]
RAW264.7 and mouse bone marrow-derived macrophages activated with anthrax lethal toxin	50 μM	Treatment inhibits pyroptosis, IL-1β maturation for the NLRP1b, NLRP3, NAIP5/NLRC4, and AIM2 inflammasomes, without affecting caspase-1 enzymatic activity—an effect not altered by ROS scavengers (NAC)—and the NLRP3 inflammasome in an Nrf2-independent manner (*Nrf2 (−/−)* studies).	Greaney et al. [98]
Human THP-1-derived macrophages and primary human PBMC-derived macrophage with a *Staphylococcus aureus* infection	10 µM	Treatment suppresses *S. aureus*-induced transcriptional expression of genes coding for the pro-inflammatory cytokines IL-1β, IL-6, and TNF-α, as well as for the M1 markers C-CR7, IL-23, and iNOS, and inhibits p38 and JNK phosphorylation.	Deramaudt et al. [99]
THP-1 macrophages treated with Aβ1-42	5 μM	Treatment inhibits the induced intracellular Ca^2+^ level, rescues the decrease in MerTK expression by blocking NF-κB nuclear translocation, and decreases IL-1β and TNF-α production upon Aβ1-42 stimulation. This effect is abolished by the siRNA-mediated knockdown of MerTK.	Jhang et al. [100]
Primary human T-cells from healthy donors or patients with rheumatoid arthritis	5 or 10 μM	Treatment inhibits the activation of untransformed human T-cells and downregulates the expression of the transcription factor RORγt and T_H_17-related cytokines (IL-17A, IL-17F, and IL-22); this effect is reversed by exogenously supplied GSH and by treatment with NAC.	Liang et al. [101]
PMA- and a23187 (PMACI)-stimulated human mast cells (HMC-1 cells)	0.1, 1, or 10 μM	Treatment inhibits the levels of inflammatory mediators including TSLP, TNF-α, IL-1β, IL-6, and IL-8, suppresses the translocation of NF-κBp65 into the nucleus and the phosphorylation of IκBα in the cytosol, and downregulates the phosphorylation of MAPK.	Jeon et al. [102]

Abbreviations: see Table 5.

**Table 2:** Anti-inflammatory effect of sulforaphane via the modulation of astrocytes and glial cells.

Cellular Model and Treatment	Concentration	Key Findings	Reference
LPS-stimulated mouse microglial BV2 cells	5 µM	Treatment improves mitochondrial impairment and neuroinflammation (levels of IL-1β, TNF-α, and NF-κB activity)—an effect dependent on HO-1 induction (confirmed by the inhibitor and the sRNA of Nrf2 studies).	Brasil et al. [103]
EOC-20 microglial cells treated with Aβ oligomers	5 µM	Treatment reverses the decrease in phagocytic (fluorescent latex beads) activity.	Chilakala et al. [104]
LPS-activated N9 murine microglial cells	5 µM	Treatment induces the translocation of Nrf2 to the nucleus and activates the ERK1/2 pathway. The siRNA-mediated knockdown of Nrf2 partly abolishes the reduction in ROS, NO, and pro-inflammatory cytokines (TNF-α, IL-1β, and IL-6), induces the Mox phenotype, inhibits microglia-mediated neurotoxicity (SH-SY5Y cells), suppresses the induced expression of miRNA and miR-155 expression, and inhibits the NF-κB, c-Fos, and c-Jun subunits of AP-1 activities.	Eren et al. [105]
Primary astroglial cultures of rat or mouse cerebral cortices	10 μM	Treatment suppresses ROS and NO production after glutathione depletion and increases *HO-1* gene expression.	Iizumi et al. [106]
Primary cultures of cortical astrocytes from the newborn pig brain treated with TNF-α and an excitotoxic glutamate	1 µM	Treatment inhibits Nox4 activity, reduces ROS production, and suppresses apoptosis.	Liu et al. [107]
Senescent astrocytes isolated from Wistar newborn rats	1 μM	Treatment decreases IL-1α secretion while increasing IL-10.	Maciel-Barón et al. [108]
LPS-stimulated primary glial cell cultures	ITH12674—melatonin-sulforaphane hybrid—10 μM	Treatment reduces inflammatory markers, NO release, and iNOS expression, suppresses IL-1β and TNFα release, and increases the Nrf2-dependent enzymes (GCLM and HO-1). The effect is Nrf2-dependent, as evidenced by Nrf2 knockout (*NRF2^−/−^*), but not totally abolished. It also prevents NF-κB translocation and reduces the overexpression of P-p38 and the binding of LPS to the TLR4/MD2 dimer.	Michalska et al. [109]
BV2 microglial cells treated with MGO-derived AGEs	5 or 10 µM	Treatment inhibits the formation of MGO-AGEs, suppresses the production of ROS, iNOS, and COX-2 and NLRP3 protein expression, lowers the expression levels of the AGE receptor (RAGE), inhibits GSK3β activation and p38 phosphorylation (but not ERK and JNK phosphorylation), and inhibits NF-κB activation/translocation and cytokine (TNF-α and IL-6) production.	Subedi et al. [110]
LPS-activated primary co-cultures of rat microglial and astroglial cells	1–15 µM	Treatment suppresses the release of TNF-α, IL-1β, IL-6, and NO, increases the mRNA level and the activity of NQO-1, and increases the cellular glutathione content.	Wierinck et al. [111]
LPS-stimulated primary cultured microglia	30 µM	Treatment reduces the mRNA levels of TNF-α and IL-1β while increasing IL-10—an effect abolished by Akt inhibition and also conformed in vivo.	Wu et al. [112]
BV-2 microglia stimulated by LPS	5–15 µM	Treatment suppresses TNF-α, IL-1β, IL-6, and iNOS and blocks MAPKs (p38, JNK) and NF-κB p65.	Qin et al. [113]
Müller cells (glial cells found in the human retina) exposed to 25 mM glucose	2.5 µM	Treatment reduces the generation of pro-inflammatory cytokines (TNF-α, IL-6, and IL-1β), enhances the activity of antioxidant enzymes (GSH, SOD, and CAT) and the nuclear accumulation of Nrf2, and increases the expression of HO-1 and NQO1.	Li et al. [51]
Primary rat microglia and the murine microglia cell line BV2 stimulated by LPS	1 µM	Treatment decreases NO production and inhibits the induced ERK1/2 and JNK phosphorylation and NF-κB and AP-1 activation.	Brandenburg, et al. [114]
LPS-activated BV2 microglia cells	5 or 10 µM	Treatment inhibits NO production and iNOS and COX-2 expression, the phosphorylation of JNK, ERK, and p38, NF-κB and AP-1, and the production of pro-inflammatory cytokines (IL-6, TNF-α, IL-1β) and PGE2 and increases Nrf2 and HO-1.	Subedi et al. [115]
C6 astrocyte cell line stimulated with LPS	5 µM	Treatment increases the mRNA levels of HO1, suppresses NADPH oxidase activity while enhancing SOD activity and the glutathione metabolism, suppresses the mRNA expression of TNF-α, IL-1β, p65 NF-κB, COX-2, and iNOS, increases the IL-10 level, suppresses TLR (mRNA) expression and NOX activity, reduces the ROS levels while increasing the activities of SOD, CAT, and GPx, GCL activity, GCL mRNA expression, and the GSH levels—an effect dependent on HO-1 (inhibitor studies).	Bobermin et al. [116]

Abbreviations: see Table 5.

**Table 3:** Anti-inflammatory effect of sulforaphane via the modulation of endothelial cells.

Cellular Model and Treatment	Concentration	Key Findings	Reference
HUVECs treated with serum from patients with severe COVID-19	1 µM	Treatment abolishes increased ROS generation via enhancing Nrf2 activity and partially restores the reduced NO level.	Rodrigues et al. [117]
Angiotensin II-mediated HUVEC injury	2 μM	Treatment inhibits oxidative stress and mitochondria-related apoptosis—effects mediated via Nrf2.	Zhang et al. [118]
AGE-stimulated HUVECs and -i rat aorta	1.6 μM	Treatment suppresses induced MCP-1, ICAM-1, and VCAM-1 gene expression and inhibits THP-1 cell adhesion to activated HUVECs, oxidative stress generation, and NADPH oxidase activation.	Matsui et al. [119]
LPS-stimulated HUVECs	1, 10, or 20 µM	Treatment prevents induced ICAM-1 and VCAM-1 expression, inhibits the induced phosphorylation of STAT3—an effect similar can be obtained with the STAT3 inhibitor (Stattic) or the STAT3 small interfering RNA— and suppresses THP-1 monocyte adhesion to activated HUVECs.	Cho et al. [120]
TNF-α-stimulated HUVECs	0.5–8 μM	Treatment suppresses MCP-1, IL-8, soluble VCAM-1, and soluble E-selectin production and inhibits NF-κB transcriptional activity, IκBα degradation, NF-κB p65 nuclear translocation, and monocyte adhesion to activated HUVECs.	Nallasamy et al. [121,122]
PMA-, TNF-α-, IL-1β-, and caecal ligation-stimulated HUVECs	5–30 μM	Treatment inhibits the induced endothelial cell protein C receptor (EPCR) shedding and the expression and activity of PMA-induced TACE and reduces the induced phosphorylation of p38, ERK 1/2, and JNK.	Ku et al. [123]
ECV304 endothelial cells stimulated with TNF-α	2.5–10 µM	Treatment inhibits the expression of ICAM-1, the production of IL-1β, IL-6, and IL-8, the phosphorylation of IκB kinase (IKK) and IκBα, Rho A, ROCK, ERK1/2, and the plasminogen activator inhibitor-1 levels.	Ku and Bae [124]
HUVEC treated with TNF-α	10–50 μM	Treatment inhibits the production of thrombin and FXa, thrombin-catalysed fibrin polymerisation, and platelet aggregation and suppresses the activity of thrombin and FXa.	Ku and Bae [124]
LPS-stimulated ECV304	10 μM	Treatment inhibits the translocation of NF-κB into the nucleus, decreases the phosphorylation of ERK, JNK, and p38 MAPK—a main effect via p38 MAPK and JNK (confirmed by gene blockade studies)—and downregulates the LPS receptor (TLR-4).	Shan et al. [125]
TNF-α-stimulated HUVECs and human aortic endothelial cells	10 μM	Treatment inhibits the induced expression of endothelial lipase expression (mRNA and protein), the induced phosphorylation of IκB kinase (IKK) 1/2 and IκB-α, and the binding of NF-κB to binding sites in the *LIPG* gene.	Kivelä et al. [126]
Human saphenous vein endothelial cell hypoxia-reoxygenation model	5 μM	Treatment increases Nrf2 protein expression, SOD activity, and the mRNA levels of *SOD1/2* and *NQO-1* and suppresses p65 and p-p65 expression and the level of TNF-α, IL-1β, IL-6, and *MCP-1* mRNA; this effect is dependent on Nrf2 (knockout studies).	Fukunaga et al. [127]
Human brain endothelial cell line (HBMEC-3)	10 µM	Treatment suppresses E-selectin and VCAM-1 expression, activates Nrf2 and its nuclear translocation, and suppresses ROS production.	Holloway et al. [128]
Human aortic endothelial cells	1–4 μM	Treatment suppresses TNF-α-induced MCP-1 and VCAM-1 mRNA and protein levels but not ICAM-1 expression, and it inhibits the induced activation of p38 MAPK, but not JNK; this effect is not mediated via Nrf2 expression.	Chen et al. [129]

Abbreviations: see Table 5.

**Table 4:** Anti-inflammatory effect of sulforaphane via the modulation of epithelial cells.

Cellular Model and Treatment	Concentration	Key Findings	Reference
LPS-stimulated primary goat mammary epithelial cells	1.25–5 µM	Treatment suppresses *TNF-α*, *IL-1β*, and *IL-6* mRNA levels and the protein levels of COX-2 and iNOS, downregulates the phosphorylation levels of the IκBα and NF-κB p65 proteins, suppresses the ROS level while increasing the levels of the expression of phase II detoxifying enzymes including HO-1, NQO1, GCLC, and GCLM, induces autophagy, and promotes autophagosome formation.	Shao et al. [130]
Bronchial epithelial IB3-1 cells exposed to the SARS-CoV-2 spike protein (*S*-protein)	5 or 10 µM	Treatment inhibits mRNA and protein-level expression of *IL-6* and *IL-8*; other cytokines and chemokines inhibited in terms of their protein level are PDGF, IL-9, G-CSF, GM-CSF, IFN-γ, MCP-1, and MIP-1β.	Gasparello et al. [131]
Human bronchial epithelial cells exposed to particulate matter PM2.5	1–5 µM	Treatment suppresses ROS production and MDA level, improves cell viability, suppresses inflammatory mediator (IL-6 and IL-8) production, and increases the nuclear levels of Nrf2 and the cytoplasmic levels of HO-1.	Qin et al. [132]
Human retinal pigment epithelial (ARPE-19) cells exposed to PM_2.5_	1 μM	Treatment improves cell viability, and reduces the ROS level, enhances SOD and CAT activities, and increases cell survival factor serum- and glucocorticoid-inducible kinase 1 (SGK1).	Sim et al. [133]
Human retinal pigment epithelial (ARPE-19) cells exposed to blue light	5 μM	Treatment improves cell viability; reduces oxidative stress, activates Nrf-2, HO-1, and thioredoxin-1, enhances the GSH levels—an effect abolished by the Nrf2 inhibitor (ML385)—inhibits ICAM-1 expression also induced by TNF-α,blocks NF-κB p65 nuclear translocation, and increases the protein expression of SIRT1 and PGC-1α gene expression.	Yang et al. [134]
LPS-treated Caco-2 cells	0.5, 1 or 5 μM	Treatment increases cell viability and abolishes apoptosis, reduces the ROS level, increases antioxidants (SOD, GPx, CAT, and total antioxidant capacity), suppresses the level of inflammatory cytokines (IL-1β, IL-6, IL-8, and TNF-α), and upregulates phosphorylated AMPK, SIRT1, and PGC-1α.	Zhang and Wu [135]
Human lung epithelial cells (BEAS-2B) exposed to cigarette smoke extract and particulate matter	10 μM	Treatment suppresses ROS and cytokines and chemokines production such as IL-6, IL-8, IL-1β, MCP-1, TNF-α, and CXCL-1; it also enhances the nuclear transcriptional activity of Nrf2 as well as the mRNA levels of downstream genes (*NQO1*, *HO-1*, *TXN*, and *TXNRD and suppresses the* phosphorylation levels of ERK and JNK.	Son et al. [136]
Human bronchial epithelial (BEAS-2B) cells exposed to diesel exhaust particles	10 µM	Treatment activates (mRNA and genes) the *Nrf2*, *NQO1*, and *HO-1* and increases autophagy marker p62 and LC3B through an Nrf2-mediated response (siRNA studies).	Frias et al. [137]
Human sinonasal epithelial cell derived from patients stimulated by house dust mite	10 μM	Treatment reverses epithelial cell junction protein ZO-1 and a decrease in transepithelial electrical resistance.	London et al. [138]
Primary mouse and tracheal and human bronchial epithelial cells sensitised by allergens (house dust mite (HDM) or *Alternaria alternata* (ALT))	10–30 μM	Treatment suppresses IL-33, IL-17E, and IL-1α levels (also in vivo) and inhibits the activation of DUOX1, ROS formation, and EGFR activation.	Danyal et al. [139]
Human mammary epithelial (MCF-10A) cells stimulated by TPA	2.5, 12.5, or 25 μM	Treatment inhibits the expression of *COX-2* (protein and mRNA), which was NF-κB-dependent, inhibits NF-κB (by modulating the phosphorylation and the subsequent degradation of IκBα) and IκB kinase (IKK) activation—effects confirmed by transfection and specific siRNA studies. The TPA effect is mediated by ERK1/2 signalling, which is inhibited.	Kim et al. [140]
Retinal pigment epithelial (RPE) 19 cell exposed to H_2_O_2_	10 μM	Treatment enhances cell viability and gene (microarray mRNA) expression of *NQO1*, sulphiredoxin 1 homolog (*SRXN1*), *GCLM*, the thioredoxin-interacting protein (*TXNIP*), *CCL2*, bradykinin receptor B1, *TXN 1*, and transcription factor Nrf2, upregulates antioxidant enzymes (NQO1; SRXN1, GCLM, Trx1, and SRXN1), and enhances the nuclear translocation of Nrf2.	Ye et al. [141]
Human nasal epithelial (HEK293T) cell exposed to influenza A virus	1 μM	Treatment decreases viral entry and replication and increases antiviral mediators/responses—RIG-I, IFN-β, and MxA—at the baseline, in the absence of infection. There is an inverse relationship between Nrf2 expression and viral entry/replication.	Kesic et al. [142]
BEAS-2B cells exposed to cigarette smoke extract	5 μM	Treatment enhances the translocation of Nrf2, increases the Nrf2-dependent gene expression of *NQO1*, *GCLM*, and *HO-1*, and inhibits IL-8 and MCP-1 production.	Starrett et al., 2011 [143]
Human airway epithelial (NCI-H292) cells	10–30 µM	Treatment downregulates MUC5AC synthesis by inhibiting ROS generation and augmenting leukocyte proteinase inhibitor production—an Nrf2-dependent effect (confirmed via an siRNA study).	Qi et al. [144]
Airway epithelial (BEAS-2B) cells stimulated by diesel particles	0.3–6.25 µM	Treatment increases phase II enzyme genes GSTM1 and NQO1, increases GST activity, and suppresses IL-1β, IL-8, and GM-CSF.	Ritz et al. [145]
LPS-stimulated HepG2 cells	2 μM	Treatment suppressed IL-6 and hepcidin production.	Al-Bakheit et al. [146]

Abbreviations: see Table 5.

**Table 5:** Anti-inflammatory effect of sulforaphane via the modulation of other cell types.

Cellular Model and Treatment	Concentration	Key Findings	Reference
Mouse C2C12 embryonic myoblasts treated by LPS	1–10 μM	Treatment reduces IL-1β secretion, ROS production, and the levels of TLR4, NLRP3, apoptosis-associated speck-like protein, and Caspase-1.	Wang et al. [147]
C2C12 myotubes in palmitic acid-induced oxidative stress and inflammation	5–10 μM	Treatment suppresses IL-6 and TNF-α, enhances Nrf2)/haem oxygenase-1(HO-1) pathway protein, and suppresses CX3CL1 and CX3CR1 expression.	Faridvand et al. [148]
VSMCs stimulated by TNF-α	5 μM	Treatment inhibits IκBα degradation and NF-κB p65, ICAM-1 mRNA, andVCAM-1, p65 (and translocation), and GATA6 expression, and reduces the binding of GATA6 to the VCAM-1 promoter.	Kwon et al. [149]
Cultured mouse vascular smooth muscle cell lines stimulated by TNF-α	8.5–42.6 μM	Treatment inhibits ROS production and the activation of p38, ERK, and JNK, inhibits NK-κB, AP-1, ΙκΒ kinase activation, the degradation of ΙκΒα, and the nuclear translocation of p65 NF-κB, decreases the c-Jun and c-Fos protein levels, and inhibits VCAM-1 expression.	Kim et al. [150]
Oxyhaemoglobin-induced inflammation in rat VSMCs	5 μM	Treatment enhances the activity of the Nrf2-ARE pathway and suppresses cytokine (IL-1β, IL-6, and TNF-α) release.	Zhao et al. [151]
Chondrocytes from patients with knee osteoarthritis stimulated with IL-1β or TNF-α	5 μM	Treatment inhibits *mPGES*, *COX-2*, and *iNOS* at the mRNA and protein levels and proteoglycan and type II collagen degradation products’ release in explant cultures and inhibits the production of PGE2 and NO.	Kim et al. [152]
Primary human articular chondrocytes, in fibroblast-like synovial cells and the SW-1353 cell line stimulated with IL-1	10 μM	Treatment attenuates NF-κB signalling at least through the inhibition of DNA binding—cytokine-induced destruction of bovine nasal cartilage at both the proteoglycan and collagen breakdown levels. Nrf2 knockdown reduces *HMOX1* expression but not *MMP1* expression, induces the phosphorylation of JNK and p38 MAPK, and inhibits the transcription of NF-κB.	Davidson et al. [153]
LPS-treated retinal pigment epithelial (ARPE-19) cells	5–30 μM	Treatment downregulates PWRN2 and inhibits NF-κB activation.	Song et al. [154]
Synoviocytes treated with TNF-α	2.5 μM	Treatment inhibits NF-κB activity and IL-1β and IL-6 secretion, activates Nrf2, and induces apoptosis in TNF-α-activated synoviocytes.	Fragoulis et al. [155]
Human embryonic kidney 293T (HEK293T) cells transfected with NOD2	5 or 10 μM	Treatment suppresses ligand-induced NF-κB activity. Note: NOD2 functions as an intracellular PRR for muramyl dipeptide.	Folkard et al. [156]
IL-1β-induced proliferation of rheumatoid arthritis synovial fibroblasts	20 µM and higher	Treatment inhibits cell proliferation and the induced expression of *MMP-1*, *MMP-3*, and *COX-2* mRNA and proteins and suppresses PGE2 production, the phosphorylation of ERK-1/2, p-38, and JNK, and the activation of NF-κB.	Choi et al. [157]
Mouse pancreatic acinar cells	10 μM	Treatment increases Nrf2 expression and Nrf2-regulated redox genes (NQO1, HO-1, SOD1, and GPx1), suppresses the cerulein-induced activation of the NLRP3 inflammasome and suppresses NF-*κ*B activation and modulated NF-*κ*B-responsive cytokine (TNF-α, IL-1β, and IL-6) expression (mRNA).	Dong et al. [158]
Ex vivo human full-thickness skin combined with in vitro HaCaT keratinocytes—UV exposure	5 or 10 μM	Treatment increases Nrf2 activity and Nrf2-dependent gene expression (*GCLM*, *HO-1*, *NQO1*) and reverses the reduced level of CAT, cell death, and structural damage.	Ernst et al. [159]
HaCaT, human keratinocyte cells activated by IFN-γ and TNF-α	10 or 20 μM	Treatment inhibits induced NF-κB and STAT1 activation and suppresses induced TARC/CCL17 and MDC/CCL22 production through the induction of HO-1 (effect completely abolished by HO-1 siRNA).	Jeong et al. [160]
Hydrogen peroxide-stimulated human neuroblastoma SH-SY5Y cells	5 μM	Treatment reduces the secretion of IL-1β and TNF-α, as well as the levels of COX-2, and decreases the activity of NF-κB and the p65 NF-κB subunit in the cell nucleus—an effect abolished by the HO-1 inhibitor and the silencing (siRNA) Nrf2.	de Oliveira et al. [161]
N2a/APPswe cells—cellular model of AD	1.25 or 2.5 μM	Treatment decreases the levels of Aβ_1-40_ and Aβ_1-42_, reduces the level of ROS, IL-1β, and IL-6, increases SOD, reduces phosphorylated NF-κBp65 COX-2 (and the iNOS protein), upregulates the expression of Nrf2 and its nuclear translocation, and decreases the DNA demethylation levels of the Nrf2 promoter.	Zhao et al. [162]

Abbreviations for Table 1, Table 2, Table 3, Table 4 and Table 5: Aβ, amyloid beta; Akt, protein kinase B; AIM2, absent in melanoma 2; AGEs, advanced glycation end products; AMAP, AMP-activated protein kinase; AP-1, activator protein 1; ARE, antioxidant-responsive element; CAT, catalase; C/EBP, CCAAT/enhancer-binding proteins; COX-2, cyclooxygenase-2; CCL2, chemokine (C-C motif) ligand 2; CCR7, C-C motif chemokine receptor7; COPD, chronic obstructive pulmonary disease; CREB, cyclic AMP (cAMP) response element-binding protein; CXCL-1, chemokine (C-X-C motif) ligand 1; CX3CR1, CX3C motif chemokine receptor 1; DCs, dendritic cells; *GCLC*, glutamate–cysteine ligase, catalytic subunit; DNMT3a, DNA (cytosine-5)-methyltransferase 3A; EGFR, epidermal growth factor; ERK, extracellular-regulated kinases; *GATA6*, GATA-binding factor 6; GCLM, glutamate–cysteine ligase modifier subunit; GPx, glutathione *peroxidase*; GR, glutathione reductase; GM-CSF, GSH, glutathione (reduced form); HDAC2, histone deacetylase 2; GSK3β, glycogen synthase kinase-3β; GST, glutathione transferase; *GSTM1*, glutathione *S*-transferase Mu 1; HIF-1α, *hypoxia-inducible factor*; HLA-DR, human leukocyte antigen DR AChain; *HMOX1*, haeme oxygenase 1 gene; HO-1, haeme oxygenase-1; ICAM-1, intercellular adhesion molecule-1; IκB, inhibitor of nuclear factor κB; IFN-β/γ, interferon-β/γ; IKK, inhibitor of nuclear factor-κB kinase; IL, iNOS, *inducible nitric oxide synthase*; JNK, c-Jun N-terminal *kinases*; LPS, MALP-2, macrophage-activating lipopeptide-2; *MAPK*, mitogen-activated protein *kinase*; MRK, MAPK kinase; MRC1, mannose receptor C-Type 1; MCP-1, *monocyte chemoattractant protein-1*; MEK-1/2; MerTK, Mer tyrosine kinase; miR, microRNA; MIP-1β, macrophage inflammatory protein-*1*β; MDA, *malondialdehyde*; MDC/CCL22, macrophage-derived chemokine; MGO, methylglyoxal; MMP, *matrix* metalloproteinases; mPGES, microsomal prostaglandin E synthase; MUC5AC, Mucin 5AC; MyD88, myeloid differentiation factor 88; NAC, *N*-Acetyl cysteine; NADPH, nicotinamide adenine dinucleotide phosphate; *NAIP5*, NLR family, apoptosis-inhibitory protein 5; NF-κB, nuclear factor-κB; NK cells, natural killer cells; *NLRC4*, NLR family caspase recruitment domain-containing protein 4; NLRP3, nucleotide-binding domain leucine-rich repeat-containing family, pyrin domain-containing 3; Nox4, NADPH oxidase 4; NO, nitric oxide; NOD2, nucleotide-binding oligomerisation domain-containing protein 2; NQO1, NADPH-quinone oxidoreductase 1; Nrf2, nuclear factor erythroid 2-related factor 2; PBMCs, PD-L1, programmed death-ligand 1; PDGF, platelet-derived growth factor; PGC-1α, peroxisome proliferator-activated receptor-gamma coactivator-1α; PGE2, prostaglandin E2; PI3K, phosphoinositide 3-kinases; PM2.5; particulate matter 2.5 µM; PMA, *phorbol*-12-myristate-13-acetate; PRR, pattern recognition receptor; RAGE, receptor for advanced glycation end products; *PWRN2*, Prader–Willi region nonprotein-coding RNA 2; RORγt, retinoic acid-related orphan receptor gamma-t; ROS, reactive oxygen species; siRNA, small interfering RNA; SOD, *superoxide dismutase*; STAT1/3, *signal transducer and activator of transcription 1/3*; TACE, tumour necrosis factor-α-converting enzyme; TARC/CCL17, thymus- and activation-regulated chemokine; TLR, toll-like receptor; TNF-α, tumour necrosis factor-α; TRAF3, tumour necrosis factor receptor-associated factor 3; TRAM, TRIF-related adaptor molecule; TRIF, TIR domain-containing adaptor molecule; TSLP, thymic stromal lymphopoietin; *TXN*, thioredoxin; *TXNRD*, thioredoxin reductase; VCAM-1, vascular cell adhesion molecular-1; VSMCs, vascular smooth muscle cells; and ZO-1, zonula occludens-1.

## Data Availability

Not applicable.
